# Genetic alterations in seborrheic keratoses

**DOI:** 10.18632/oncotarget.16698

**Published:** 2017-03-30

**Authors:** Barbara Heidenreich, Evygenia Denisova, Sivaramakrishna Rachakonda, Onofre Sanmartin, Timo Dereani, Ismail Hosen, Eduardo Nagore, Rajiv Kumar

**Affiliations:** ^1^ Division of Molecular Genetic Epidemiology, German Cancer Research Center, Heidelberg, Germany; ^2^ Department of Dermatology, Instituto Valenciano de Oncologia, Valencia, Spain; ^3^ German Consortium for Translational Cancer Research (DKTK), German Cancer Research Center, Heidelberg, Germany

**Keywords:** exome-sequencing, seborrheic keratosis, skin cancer, somatic mutations

## Abstract

Seborrheic keratoses are common benign epidermal lesions that are associated with increased age and sun-exposure. Those lesions despite harboring multiple somatic alterations in contrast to malignant tumors appear to be genetically stable. In order to investigate and characterize the presence of recurrent mutations, we performed exome sequencing on DNA from one seborrheic keratosis lesion and corresponding blood cells from the same patients with follow up investigation of alterations identified by exome sequencing in 24 additional lesions from as many patients. In addition we investigated alterations in all lesions at specific genes loci that included *FGFR3*, *PIK3CA*, *HRAS*, *BRAF*, *CDKN2A* and *TERT* and *DHPH3* promoters. The exome sequencing data indicated three mutations per Mb of the targeted sequence. The mutational pattern depicted typical UV signature with majority of alterations being C>T and CC>TT base changes at dipyrimidinic sites. The *FGFR3* mutations were the most frequent, detected in 12 of 25 (48%) lesions, followed by the *PIK3CA* (32%), *TERT* promoter (24%) and *DPH3* promoter mutations (24%). TERT promoter mutations associated with increased age and were present mainly in the lesions excised from head and neck. Three lesions also carried alterations in *CDKN2A*. FGFR3, TERT and DPH3 expression did not correlate with mutations in the respective genes and promoters; however, increased FGFR3 transcript levels were associated with increased FOXN1 levels, a suggested positive feedback loop that stalls malignant progression. Thus, in this study we report overall mutation rate through exome sequencing and show the most frequent mutations seborrheic keratosis.

## INTRODUCTION

Seborrheic keratoses represent one of the most common benign epidermal tumors that associate with increased age [1]. The lesions manifest clinically as acquired, solitary or multiple, well demarcated brownish papules or plaques with a verrucous surface that predominantly localize at areas of the head, neck and trunk [2, 3]. In contrast to actinic keratoses that can progress to squamous cell carcinoma *in situ* (Morbus Bowen) and squamous cell carcinoma of skin (SCC), seborrheic keratoses lack malignant potential [4]. The majority of seborrheic keratoses are monoclonal tumors, representing autonomous neoplasia resulting from clonal expansion of somatically mutated cells rather than epidermal hyperplasia [5]. Unlike many malignant tumors, seborrheic keratoses appear to be genetically stable but harbor multiple somatic alterations [6]. Despite lack of malignant potential, 89 percent of the lesions carry at least one and 45 percent more than one mutation in a well characterized oncogene [6, 7]. Frequent alterations affect *FGFR3* and *PIK3CA*, with mutations frequencies of 40-85% and 40%, respectively [3, 6, 8]. Other genes mutated in seborrheic keratoses include *HRAS*, *KRAS*, *EGFR* and *AKT1* [3, 6, 9]. Activation of FGFR3 appears to be a common feature in the lesions that can to some extent be attributed to *FGFR3* mutations [8, 10]. Seborrheic keratosis, despite being hyper-proliferative remain well differentiated and rather than senescence due to oncogenic signals, a positive feedback loop between FGFR3 and the transcription factor FOXN1 has been suggested to prevent malignant progression of those lesions [6, 10, 11].

As well-accessible benign tumors of the skin, seborrheic keratoses present a suitable model, which could allow an insight into the genetic changes that distinguish those lesions from neoplasia with malignant potential [2, 12]. To characterize and investigate the presence of recurrent mutations, we performed exome sequencing of DNA from one seborrheic keratosis lesion and corresponding blood cells. Follow-up sequencing of non-synonymous somatic alterations identified through exome sequencing was performed on 24 lesions. We also investigated seborrheic keratoses for alterations in genes that play a role in the development (*FGFR3, PIK3CA, HRAS*) and or those that are frequent in other skin neoplasms (*BRAF, CDKN2A*). The analysis also included sequencing of the promoter regions of the *TERT* as well as the *DPH3* gene, which are mutated at high frequencies in skin cancers [13–15].

## RESULTS

### Whole-exome sequencing

**Figure 1:**
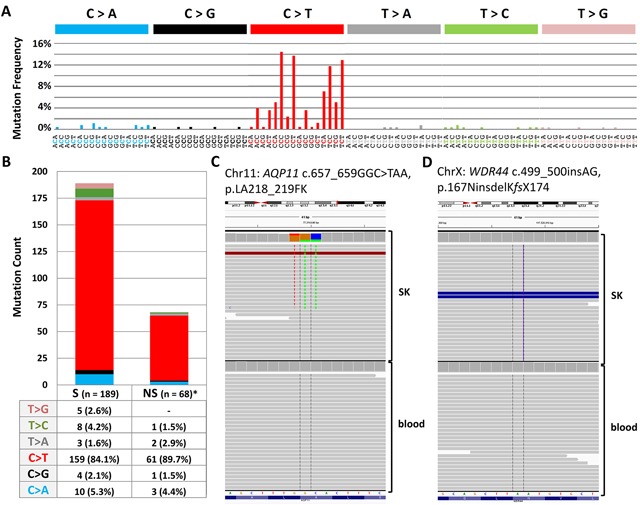
**(A)** Mutational signature from exome sequencing data dominated by characteristic UV-signature mutations at dipyrimidinic sites. **(B)** Proportion ofnon-synonymous versus synonymous mutations from exome exome sequencing **(C)** Integrative Genomics Viewer screenshots of a somatic trinucleotide mutation in *AQP11* **(D)** Integrative Genomics Viewer screenshots of a 2 bp insertion in *WDR44* that results a stop codon after 10 (KMCLKLKQKY) residues.

Exome sequencing was carried out on DNA extracted from a pathologically confirmed seborrheic keratosis and corresponding blood tissue from a 49-year old women diagnosed with melanoma. The melanoma was removed surgically and the patient was free of disease at time of removal of the seborrheic keratosis lesion. The lesion was located at left lower scapula, a self-reportedly area of intermittent sun exposure with previous history of sunburns. Exome sequencing resulted in mean target coverage of 81X for the DNA from the lesion and 60x for the DNA from blood, with 90% of bases covered at least 14-fold and 8-fold, respectively. A total of 230 somatic mutations were detected, 3 mutations per Mb of the targeted sequence (Supplementary Table 1). The mutations included 202 single nucleotide variations (78.6%), 26 tandem dinucleotide substitutions (each counted as 2; 20.2%) and one trinucleotide mutation in the *aquaporin 11* (*AQP11*) gene (Figure 1). In addition, a 2-basepair frameshift insertion in the *WDR44* gene was detected (Figure 1). Over 90% of mutations were present with an allele frequency of 20%. Of the 257 mutations, 92 were located in coding regions with 68 as non-synonymous and 24 synonymous. Non-synonymous to synonymous ratio was 2.83:1. 168 (83%) single nucleotide variations were cytidine to thymidine (C>T) transitions, with 164 (97.6%) located at dipyrimidinic sites. Additionally, 25 of the 26 dinucleotide substitutions were CC>TT changes (counted as single mutations: 50/257, 19.5%; counted as events: 25/231, 10.8%).

59 missense and nonsense variants supported by at least 20 sequencing reads were validated by Sanger sequencing. Those included 47 single nucleotide mutations, 10 tandem dinucleotide mutations, one trinucleotide mutation in *AQP11* and an insertion in *WDR44* (Supplementary Table 2). Somatic nature was confirmed by the absence of mutations in DNA from the corresponding blood by Sanger sequencing. The mutations identified also included c.1955A>T, p.K652M alterations in FGFR3 and an intronic single nucleotide variation in AKT that did not impact splicing as assessed by Human Splicing Finder and ANNOVAR.

### Mutations in additional seborrheic keratosis lesions

We further investigated 24 pathologically confirmed seborrheic keratoses excised from the same number of individuals (Supplementary Table 3). Sixteen of the 24 seborrheic keratoses were taken from patients with a previous history of skin cancer, with seven diagnosed with melanoma, 5 with BCC, 3 with SCC and one patient had a history of all three skin cancer (Supplementary Table 3). All lesions were excised from the areas of skin with chronic or intermittent sun-exposure (9 from head/neck, 14 from trunk, 1 from extremities). The DNA from the lesions was screened by Sanger sequencing for the 59 alterations identified by exome sequencing. For each mutation the sequenced region included at least 100 bp up- and down-stream. One seborrheic keratosis (SK14) carried a non-synonymous mutation in *GRIK1* (c.1148C>A, p.G383D) and an intronic mutation in *NEDD4* (c.3471-1C>T) with a potential effect on splicing (Human Splicing Finder). SK7 carried a non-synonymous mutation in *PRCC* (c.964G>A, p.E322K) and SK6 carried a synonymous mutation in *AQP11* (c.732T>G, p.S244S) (Table 1).

**Table 1:** Overview of genetic alterations in all seborrheic keratoses

Patient	Age	Sex	skin cancer	*FGFR3*	*PIK3CA*	*TERT* promoter	*DPH3* promoter	*HRAS*	*CDKN2A*
				12(48%)	8(32%)	6(24%)	6(24%)	4(16%)	3(12%)
**SK-ES^a^**	49	female	melanoma	K652M**^b^**	wt	wt	wt	wt	wt
**SK1**	69	male	BCC	wt	wt	wt	−9C>T	wt	wt
**SK2**	81	female	*no*	wt	wt	−146C>T	wt	wt	deletion**^c^**
**SK3**	78	male	*no*	wt	wt	−138_139CC>TT	wt	wt	wt
**SK4**	36	male	melanoma	K652M	wt	wt	wt	wt	wt
**SK5**	65	male	SCC	R248C	wt	−146C>T	wt	wt	wt
**SK6^d^**	81	male	BCC	R248C	E545Q**^e^**	−146C>T	wt	wt	wt
**SK7^f^**	58	male	BCC	R248C	wt	wt	wt	G12D**^g^**	wt
**SK8**	76	female	*no*	wt	E545Q	wt	wt	G12D	wt
**SK9^h^**	68	female	melanoma	wt	G1048R	wt	wt	G13V	wt
**SK10**	53	male	*no*	R248C	E542K	wt	−9C>T	wt	wt
**SK11**	64	male	melanoma	R248C	E542K	wt	wt	wt	wt
**SK12**	78	male	SCC	wt	E545Q	wt	−9C>T	wt	G106R (p14)**^i^**
**SK13**	51	female	*no*	R248C	E545Q	wt	wt	wt	wt
**SK14^j^**	84	male	SCC	wt	wt	−138_139CC>TT	wt	wt	wt
**SK15**	75	female	melanoma	wt	wt	wt	wt	wt	wt
**SK16**	65	female	BCC	K652M	wt	wt	−8C>T	wt	wt
**SK17**	86	female	BCC	wt	wt	wt	wt	wt	wt
**SK18^k^**	72	male	melanoma	wt	wt	wt	wt	wt	wt
**SK19**	73	female	melanoma	K652E	wt	wt	wt	wt	wt
**SK20**	35	male	*no*	wt	wt	wt	wt	Q61L	wt
**SK21**	75	male	melanoma	wt	E542K	−138_139CC>TT	−8_9CC>TT &-12C>T	wt	S43*fs*X**^l^**
**SK22**	69	male	melanoma	K652M	wt	wt	wt	wt	wt
**SK23**	66	male	*no*	wt	wt	wt	wt	wt	wt
**SK24**	70	male	*no*	R248C	wt	wt	−8_9CC>TT	wt	wt

^a^The lesion was analysed by exome sequencing.

^b^Nucleotide changes corresponding to R248C, c.742C>T; K652M, c.1955A>T; K652E, c.1954A>G in *FGFR3*.

^c^In addition to monoallelic deletion, the lesion carried mutation P114L, c.341C>T in *CDKN2A*.

^d^The lesion in addition had S244S (c.732T>G) mutation in *AQP11*.

^e^Nucleotide changes corresponding to E542K, c.1624G>A; E545Q, c.1633G>A; G1048R, c.3145G>C in *PIK3CA*.

^f^The lesion in addition had E322K (c.964G>A) mutation in *PRCC*.

^g^Nucleotide changes corresponding to G12D, c.35G>A; G13V, c.38G>T; Q61L, c.182A>T in *HRAS*.

^h^The patient in addition had BCC and SCC.

^i^Nucleotide change corresponding to G106R, c.316G>A in p14ARF. The amino acid change for p16 was synonymous (L91L).

^j^The lesion in addition carried G383D (c.1148C>A) mutation in *GRIK1* and intronic (c.3471-1C>T) mutation in *NEDD4* genes.

^k^The patient in addition had SCC.

^l^Nucleotide change corresponding to S43*fs*X, c.128_131DelGTTA in *CDKN2A*.

BCC = Basal Cell Carcinoma; SCC = Squamous Cell Carcinoma; *CDKN2A* = cyclin-dependent kinase inhibitor 2A; *DPH3* = diphthamide biosynthesis 3; *FGFR3* = fibroblast growth factor receptor 3; *HRAS* = Harvey rat sarcoma viral oncogene homolog; *PIK3CA* = phosphatidylinositol-4,5-bisphosphate 3-kinase, catalytic subunit alpha; *TERT* = telomerase reverse transcriptase.

### Screening for other genetic alterations

Additionally, we sequenced exon 7, 10 and 15 of *FGFR3* and detected mutations in 12 out of 25(48%) lesions (Table 1). Seven lesions carried the c.742C>T (p.R248C) mutation in exon 7 and other five in exon 15, with the c.1955A>T (p.K652M) mutation in four and one carried the c.1954A>G (p.K652E) mutation. We also detected the*PIK3CA* mutations in 8 (32%) of the lesions and *HRAS* mutations in four (16%) lesions (Table 1). None of the lesions harbored a mutation in exon 15 of *BRAF*.

We also sequenced *CDKN2A* and investigated large deletions and/or methylation at the 9p21 locus using MS-MLPA. We identified a 4 bp c.128_131DelGTTA (p.S43_Y44delinsT*fs*X51) deletion in exon 1 of *CDKN2A* in one lesion, which in addition to Sanger sequencing was confirmed by cloning of the amplified product into a T-overhang vector (Supplementary Figure 1). One lesion carried a large mono-allelic deletion at the 9p21 locus that encompassed *CDKN2A*, *CDKN2B* and *CDKN2B-AS1* promoter (chr9:21,957,523-21,985,479, hg18). Another lesion carried a mutation in the *CDKN2A* that was synonymous for p16 (c.273G>A, p.L91L) but non-synonymous for the alternate reading frame (ARF) transcript (c.316G>A, p.G106R). Overall, 3/25 (12%) lesions harbored alterations affecting *CDKN2A*.

Six (24%) of the 25 lesions carried *TERT* promoter mutations, while three had the -146C>T mutation and the other three showed the -138_139CC>TT tandem mutation. Mutations in the promoter region of the *DPH3* gene were also detected in six lesions that included three -9C>T, one -8C>T and two -8_9CC>TT alterations (Table 1).

### Somatic mutations and clinical parameters

The median age of 16 men and 9 women from whom seborrheic keratoses were excised was 69 years. The presence of the *TERT* promoter mutations in seborrheic keratoses associated with increased age; only one of the 13 lesions from patients with age 69 or lower carried the mutations, whereas 5 of 12 lesions from patients over 69 years of age carried the mutations (OR 8.6, 95% CI 0.83-89.04, p = 0.07). All seborrheic keratosis lesions with *TERT* promoter mutations originated from the head/neck (OR 61.3, 95% CI 2.76-1359.24, p = 0.009). In particular, the lesions that harbored CC>TT tandem mutations in *TERT* and *DPH3* promoter originated from head and neck, the areas of chronic sun-exposure. The mutations in *FGFR3* associated with decreased patient age and occurred more frequently in seborrheic keratosis from patients younger than 69 years (OR 6.8, 95% CI 1.16–39.20, p = 0.03); however, no difference in the *FGFR3* mutations was observed based on the localization of the lesions.

### Expression of selected genes

**Figure 2:**
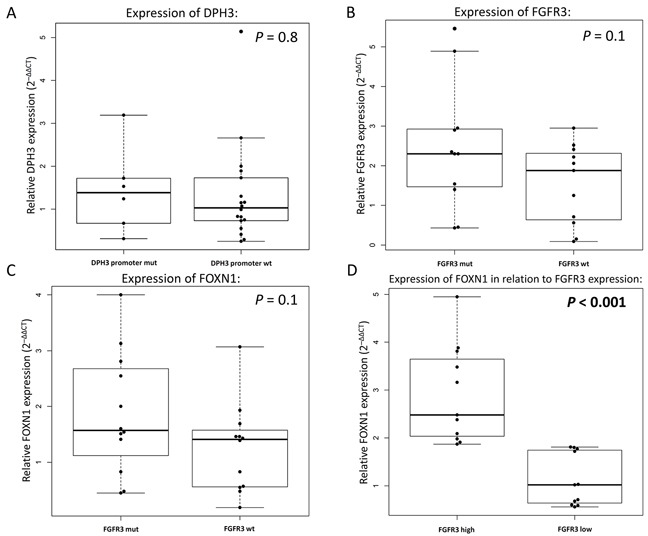
Relative gene expression in seborrheic keratosis measured by quantitative real-time PCR **(A)** Differences in the levels of DPH3 expression with and without mutations in the DPH3 promoter. **(B)** Measurement of FGFR3 expression based on presence or absence of activating FGFR3 mutations. Comparison of FOXN1 expression according to **(C)** presence of FGFR3 mutations melanoma and **(D)** expression levels of FGFR3 (according to median expression).

We measured expression of TERT, DPH3, and FGFR3 in 24 seborrheic keratoses. TERT mRNA was detected in only 6 lesions, of which four did not carry TERT promoter mutations and two carried one mutation each, -146C>T and -138_139CC>TT (Figure 2). The difference in DPH3 expression was not statistically significant between lesions with (n=6) and without (n=18) the DPH3 promoter mutations (Figure 2A). FGFR3 expression was detected in 22 lesions; however, the difference between the lesions with (n=11) and without (n=11) the *FGFR3* mutations was not statistically significant (Figure 2B). In addition to FGFR3, we measured expression of FOXN1, a transcription factor with an important role in keratinocyte differentiation and epithelial cell proliferation. Those lesions that carried FGFR3 mutations had higher levels of FOXN1 expression; however the difference was not statistically significant (Figure 2C). When seborrheic keratoses were stratified into two groups based on median FGFR3 expression (high n=11 and low n=11), the lesions with high FGFR3 expression had statistically significant increased levels of FOXN1 (Figure 2D). All the seborrheic keratosis lesions were also sequenced for the coding regions of FOXN1 and did not carry mutations.

## DISCUSSION

The skin lesions seborrheic keratoses represent benign neoplasms that harbor multiple oncogenic mutations, often at the hotspots detected frequently in malignant cancers with demonstrated impact on downstream signaling. The seborrheic keratosis, despite multiple mutations in cancer related genes, explicitly lack a malignant potential [6]. In the present study, we exome sequenced a single seborrheic keratosis, which showed high mutation prevalence with clear UV-signature and allelic frequency supporting a clonal evolution of the neoplasm. In a follow-up investigation of 24 seborrheic keratoses we did not observe any recurrence of the mutations identified through exome sequencing. Through targeted sequencing we detected previously reported mutations at a high frequency in the *FGFR3*, *PIK3CA* and *HRAS* genes. And we also report for the first time *CDKN2A* alterations in seborrheic keratoses. In a novel observation we detected noncoding mutations within the *TERT* promoter and a bidirectional promoter involving *DPH3* and *OXNAD1* at relatively high frequencies. The *TERT* promoter mutations that usually lead to an increased TERT expression are seldom present in benign tumors [16]. However, the four of the six lesions with the *TERT* promoter mutations did not show detectable level of TERT expression. Further, we detected a correlation between increased FGFR3 and FOXN1 expression levels, as an indicator of a positive feedback loop mechanism [11].

The overall mutational frequency observed through exome sequencing of a seborrheic keratosis lesion in this study ranged about 3 mutations per Mb, which is lower than that the reported frequencies of 14-111 mut/Mb in melanoma, 65-76 mut/Mb in BCC and 33-61 mut/Mb in SCC but comparable to an average mutation burden seen in many adult solid tumors [17–24]. An aged sun-exposed skin reportedly harbors low fraction of 2-6 mutations per megabase [23]. Notably, we observed high allele frequency for 90% of mutations (>20%), which is in agreement with previous reports on the clonal nature of seborrheic keratoses [5]. Skin cancers in general are not only burdened by highest number of mutations but also reflect characteristic UV signature [17–22, 24]. Most of the skin cancers in general display the typical UV mutational signature, with the exceptions of rare subtypes that arise on sun-protected parts [17, 18, 24]. The prevalence of C>T mutations at dipyrimidinic sites (164/202, 81%) in the lesions investigated in this study was higher than the numbers reported in BCC (66.7%) and SCC (67%) [17, 19]. Similarly, the number of CC>TT tandem mutations detected in the lesion exceeded BCC and SCC [18]. Our findings from a single lesion of a distinct UV-signature with high numbers of tandem mutations concur with the self-reported history of intermittent sun-exposure and previous experience of sunburns at the sampling site. Although advanced age and cumulative sun exposure are assumed as primary risk factors, the exact influence, based on reports from different study populations, of UV-light remains unclear [1, 3, 25–27].

None of the 59 missense or nonsense alteration were detected in additional 24 lesions with probable reason that either (i) those mutations were private to the sequenced exome or (ii) that affected genes carry alterations at positions other than those detected through exome sequencing [28]. Two of the affected genes, *MFSD2A* and *SLC39A1* have been reported as tumor suppressor genes in lung and prostate cancer, respectively, which by definition could carry mutations through the entire length [29–31]. Other alterations were found in genes reportedly altered in skin cancer, such as *DGKI* and *SYK* in melanoma or *BCOR*, *PIKFYVE* and *NEDD4* in SCC [18, 32, 33]. A literature search revealed that approximately one third of genes that carried non-synonymous mutations had been reported in the context of skin physiology and keratinocyte proliferation [34–41]. Activation of CDK2 in mouse epidermis reportedly induces keratinocyte proliferation, however did not affect skin tumor development [42, 43] Also SYK has been shown to act as a negative regulator in epidermal keratinocyte differentiation and is also involved in EGFR signaling, which may contribute to its regulatory role in keratinocyte terminal differentiation [44]. NEDD4 is involved in the ΔNp63α-mediated suppression of nuclear PTEN in basal layer keratinocytes, whereas nuclear PTEN inhibits cell proliferation and mice with a keratinocyte-specific null mutation of Pten reportedly exhibit epidermal hyperplasia and hyperkeratosis [45, 46]. The variety of somatic mutations found in genes with involvement in keratinocyte proliferation and/or skin cancer development leaves a room for further investigations, with follow-up studies with larger sample sizes or targeted sequencing covering complete genes.

It has been speculated that lack of malignant potential in seborrheic keratoses might be due to the absence of alterations in tumor suppressor genes [6, 7]. In this study we did detect deletions within *CDKN2A* in three lesions. Alterations of *CDKN2A* have been reported in 10-30 percent of SCC and in a limited number of actinic keratosis [47, 48]. A study on engineered skin grafts showed that FGFR3 mutants drive mild hyperplasia but are insufficient either alone or in combination with G1-S checkpoint release to cause benign or malignant epidermal tumors [49]. It has been suggested that a positive feedback loop between FGFR3 and the transcription factor FOXN1 stalls affected keratinocytes in a pro-differentiation mode and thereby prevents their malignant progression [11]. In our data we did find that high FGFR3 expressing seborrheic keratoses had high levels of FOXN1, but we did not observe increased FOXN1 expression due to activating *FGFR3* mutations [11, 49]. While some earlier reports showed association of *FGFR3* mutations with increased patient age, we observed statistically significantly increased frequency of mutations in patients below the median age. Through the presence in not only in thick but also in flat lesions, the mutations have been implied to occur at the initial appearance of seborrheic keratoses [8].

We found mutations in a bidirectional promoter for *DPH3* and *OXNAD1* genes in one-fourth of the lesions. Alterations in this promoter region were reported in SCC and BCC at frequencies approximating 40%; the functionality of these mutations however remains to be determined [14]. We also detected mutations in the core promoter of the *TERT* gene, which have been reported in different cancers including skin [13, 14, 50]. The mutations generally associate with aggressive forms of the disease and have been shown to induce TERT expression through creation of ETS transcription factor binding sites [50]. However, in contrast, the *TERT* promoter mutations in seborrheic keratosis did not result in enhanced expression and may represent sheer passenger events due to lack of requisite transcription factors in the lesions. Transgenic induction of TERT in mouse skin has been shown to cause hair follicle stem cells proliferation and activation of tissue progenitor cells through non-canonical pathways [51, 52].

In this study, we report the overall mutation rate through exome sequencing and show the most frequent mutations in seborrheic keratoses. With overall mutation rate of three per Mb, the most frequent alterations were in the *FGFR3* and *PIK3CA* genes. We also for the first time reported alterations in the *TERT* promoter, *DPH3* promoter, and *CDKN2A* gene in the lesions. FGFR3, TERT, and DPH3 expression did not correlate with mutations in the respective genes and promoters; however, increased FGFR3 transcription was associated with increased FOXN1 levels, a suggested positive feedback loop that stalls malignant progression. As we demonstrated a high proportion of UV-associated mutation, seborrheic keratoses can be considered as clinical markers of sun damage. However, it may be pointed out that the exome sequencing data presented are from a single lesion, which would, therefore, merit a cautious extrapolation.

## MATERIALS AND METHODS

### Seborrheic keratosis, blood tissues and nucleic acid extraction

Blood and fresh-frozen seborrheic keratoses tissues were retrieved from the Biobank of the Instituto Valenciano de Oncología in Valencia, Spain. Ethical approval for the study from the institutional review board and written informed consent from all study participants were obtained. DNA and RNA from fresh frozen tissues were extracted using the QIAGEN AllPrep DNA/RNA/miRNA Universal Kit (QIAGEN). Tissues had been snap-frozen in liquid nitrogen after surgical removal by shaving technique, therefore, with minimal dermal component and kept at -80°C until nucleic acid extraction. For DNA and RNA extraction, tissues were placed in 600 ml RLT buffer and homogenized in a Tissuelyser LT (QIAGEN) with 5mm stainless steel beads (5 min at 30 Hz). The homogenate was further processed following standard protocols and with separate steps for DNA and RNA extraction. Before processing the RNA-containing fraction, a clean-up step to remove lipids and fatty tissues was performed with 150 μl chloroform and centrifugation for 3 min at maximum speed at 4°C. The aqueous phase was further processed for RNA extraction. Concentrations of total DNA and RNA extracted were measured using an ultraviolet–visible spectrophotometer (NanoDrop Technologies, Thermo Fisher Scientific Inc.).

For extraction of DNA from blood, we used 20 μl volume and the QIAamp 96 DNA Blood Kit (QIAGEN). All steps were performed at room temperature. After protease treatment and lysis, DNA bound to column membrane was cleaned in several washing steps and eluted with a buffer.

### Exome capture and Illumina sequencing

Exome capture was performed using Agilent SureSelect Target Enrichment System, Human All Exon V5+UTRs kit (Agilent Technologies) according to manufacturer's protocol. Exome capture area comprised 286754 targets from 21522 genes including untranslated regions (~75Mb in total). Sequencing of DNA from the seborrheic keratosis lesion and matched normal blood sample was carried out on Illumina Hiseq2000 platform with 101-bp paired-end reads following Illumina-provided protocol. Both DNA samples were sequenced on two sequencing lanes. Coverage statistics for the capture regions was generated with Genome Analysis Toolkit (GATK) version 3.1-1 [53]. A mean coverage of x81 and x60 was obtained for DNA from the lesion and blood tissue, respectively.

### Read mapping and data preprocessing

For each sequencing lane read pairs were mapped to the human reference genome (build hg19) using Burrows-Wheeler Aligner (BWA) version 0.7.5a mem function with default parameters [54]. BAM files were coordinate-sorted and duplicates were removed by Picard software version 1.102 (see URLs). Base quality score recalibration and local realignment around indels were performed by GATK (version 2.4-9) on the lane-level data. Lane-level data from one sample were then merged into one BAM file using Picard. Lane, library and sample information was captured in the read group tag of the merged final BAM file. Second round of duplicates removal was performed on the sample level. Additional round of local realignment was performed jointly for the matched SK and normal sample to avoid alignment differences between the tissues from the same patient as suggested by GATK “best practices”. All preprocessing steps were performed for the capture regions with 50 bp padding.

### Somatic variant calling

Capture regions with 50 bp padding (~98 Mb in total) were used for variant calling to include flanking non-coding regions. Somatic single nucleotide variants were detected by Mutect algorithm [55]. The minimum base quality of 30 was required. Candidates with at least one high-quality base supporting alternate allele in the patient-matched normal sample were excluded. Mutations marked as “KEEP” were used in further analysis. Coverage cutoffs of at least 14 reads in the lesion and at least 8 reads in the normal applied in Mutect resulted in ~88 Mb of “callable” bases which were used to estimate the somatic mutation frequency. Short insertions/deletions (indels) were called by GATK Unified Genotyper in both lesion and normal samples. Somatic indels in the lesion were identified by filtering with a list of alterations called in the normal sample. Variant annotation was performed by ANNOVAR [56] using RefSeq genes annotations [57], dbSNP (Build ID: 137), variants from 1000 Genomes project and Catalogue of Somatic Mutations (COSMIC) version 67 [58, 59]. All variants were manually reviewed using Integrative Genomics Viewer [60]. Several indels were excluded due to the low coverage, poor alignment quality and overlapping repetitive regions. New single somatic nucleotide variations identified by manual inspection are marked as such in the corresponding table (Supplementary Table 1). Missense and nonsense variants supported by at least 20% of sequencing reads were selected for validation by Sanger sequencing.

### Mutational analysis by PCR and sanger sequencing

Mutations at different loci were screened by PCR and Sanger sequencing. Each PCR was carried out in 10 μl volume containing 10 ng DNA, 0.11 mM dNTP and 0.15 μM of each primer and Taq polymerase (GENAXXON biosciences GmbH). Concentrations of MgCl_2_ and further additives as well as annealing temperature were adjusted according to primer sequences (Supplementary Table 4). Amplified products were purified with ExoSAP (illustra ExoProStar, GE Healthcare Life Sciences) to remove unused primer and were subjected to 35 cycles of sequencing reaction with a di-deoxy terminator kit and forward and reverse primers in separate reactions (BigDyeTerminator v3.1 Cycle Sequencing Kit, life technologies, Thermo Fisher Scientific Inc.) and analyzed in a capillary sequencer (AbiPrism 3130xl Genetic Analyzer). The sequencing data were analyzed using Geneious Pro 5.6.5 software and sequences from the NCBI (National Center for Biotechnology Information) gene database were used as references.

### Methylation-sensitive multiplex ligation-dependent probe amplification (MS-MLPA)

MS-MLPA was carried out with specific probes (SALSA MLPA ME024 9p21 CDKN2A/2B; MRC Holland, Amsterdam, The Netherlands). Fifty ng of DNA in 5μl 1% TE per reaction were subjected to 16 hours of incubation with the probe mix and then divided into two reactions. One reaction mix was processed in a ligation reaction, followed by a multiplex PCR. The second part was processed in a ligation step followed by digestion with HhaI restriction enzyme and subjected to amplification in a multiplex PCR. PCR products were subjected to fragment analysis in a capillary sequencer (AbiPrism 3130xl Genetic Analyzer) using POP-7 polymer. The results were evaluated using Coffalyser software (MRC-Holland); threshold to define deletions was set at the delta value of 0.3 and if methylation of a probe exceeded 30% the status was considered positive.

### Molecular cloning

Molecular cloning was performed to confirm a 4bp insertion or deletion indicated by Sanger sequencing. The PCR amplicon of the region of interest harboring the alleged insertion/deletion (*CDKN2A*, exon1) was cloned into a T-overhang vector (TOPO® pCR2.1, Invitrogen) and introduced into DH5α *E. coli* cells (Invitrogen, USA). Sequencing of the plasmid was performed by PCR and Sanger sequencing using M13 forward (5′GTAAAACGACGGCCAG3′) and M13 reverse (5′CAGGAAACAGCTATGAC3′) primer. Extraction of the plasmid was performed using an appropriate kit (PureLink Quick Plasmid Miniprep Kit, Invitrogen).

### Measurement of mRNA expression by quantitative real-time PCR (qRT-PCR)

For measurement of gene expression, reverse transcription reactions were performed with approximately 1.0 μg RNA and random hexamer primers using a cDNA synthesis kit (Thermo Scientific, Waltham, USA). Gene expression levels were then determined by quantitative real-time PCR using a SYBR Green kit (Qiagen). The real-time PCR was carried out in triplicates on a 384-well layout using primers specific for TERT (forward 5′-CGGAAGAGTGTCTGGAGCAA-3′; reverse 5′-GGATGAAGCGGAGTCTGGA-3′), DPH3 (Qiagen), FOXN1 (Qiagen) and FGFR3 (Qiagen) and primers for the *GUSB* gene (Qiagen), a housekeeping gene used as an internal standard. Gene expression levels were calculated using GUSB expression as a reference and relative quantification was performed using the ΔΔC_T_ method and log2 transformation.

### URLs

Burrows-Wheeler Aligner (BWA), http://bio-bwa.sourceforge.net/; Genome Analysis Toolkit (GATK), http://www.broadinstitute.org/gatk/; Picard, http://broadinstitute.github.io/picard/; MuTect, http://www.broadinstitute.org/cancer/cga/mutect; ANNOVAR, http://annovar.openbioinformatics.org/en/latest/; dbSNP, http://www.ncbi.nlm.nih.gov/SNP/; COSMIC, http://cancer.sanger.ac.uk/cancergenome/projects/cosmic/; Integrative Genomics Viewer (IGV), http://www.broadinstitute.org/igv/; R, http://www.R-project.org/; NCBI RefSeqGene, http://www.ncbi.nlm.nih.gov/refseq/rsg/; Human Splicing Finder - Aix Marseille Université, http://www.umd.be/HSF3/index.html.

## SUPPLEMENTARY MATERIALS FIGURES AND TABLES








