# Supplementary material for: Genetic alterations in seborrheic keratoses
Source: Oncotarget. 2017 Mar 30;8(22):36639–49. doi: 10.18632/oncotarget.16698 (PMC5482683; doi:10.18632/oncotarget.16698)
Supplement: Supplementary file 1 [file oncotarget-08-36639-s001.pdf]

Genetic alterations in seborrheic keratoses

SUPPLEMENTARY MATERIALS

SUPPLEMENTARY FIGURE AND TABLES

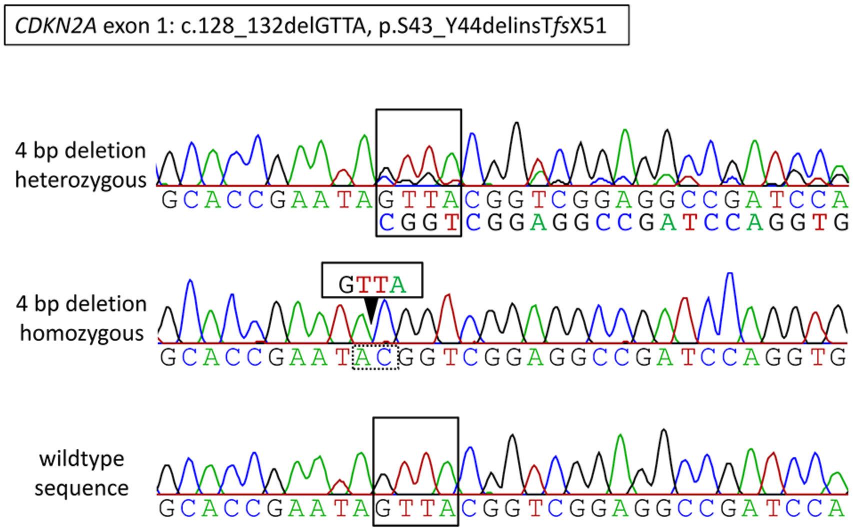

Supplementary Figure 1: Sequences showing a 4 bp deletion in exon 1 of CDKN2A in DNA from SK21 lesion as seen by initial Sanger sequencing (upper panel) and after molecular cloning (middle panel). The deletion results in a frameshift leading to premature stop codon.

Supplementary Table 1: Somatic alterations detected through whole-exome sequencing

See Supplementary File 1

Supplementary Table 2: Validated somatic variants detected by Sanger sequencing

See Supplementary File 2

Supplementary Table 3: Overview of Clinical Information and Expression Data for 25 Seborrheic Keratoses

Lesion	Age	Gender	Location	Pathology	History of skin cancer	TERT expression	FGFR3 expression
**SK-ES**[a]	49	female	trunk	pigmented	melanoma	*not detectable*	high
**SK1**	69	male	trunk		BCC	*not detectable*	high
**SK2**	81	female	head/neck		*no*	*not detectable*	*not detectable*
**SK3**	78	male	head/neck	pigmented	*no*	expression	low
**SK4**	36	male	trunk	acanthotic	melanoma	*not detectable*	high
**SK5**	65	male	head/neck		SCC	*not detectable*	*not detectable*
**SK6**	81	male	head/neck		BCC	*not detectable*	low
**SK7**	58	male	trunk		BCC	expression	low
**SK8**	76	female	extremities		*no*	*not detectable*	low
**SK9**	68	female	trunk		melanoma; BCC; SCC	-	-
**SK10**	53	male	trunk		*no*	*not detectable*	high
**SK11**	64	male	head/neck	irritated (inflamed)	melanoma	expression	high
**SK12**	78	male	trunk	pigmented	SCC	*not detectable*	low
**SK13**	51	female	trunk		*no*	*not detectable*	high
**SK14**	84	male	head/neck	pigmented	SCC	expression	low
**SK15**	75	female	trunk		melanoma	*not detectable*	low
**SK16**	65	female	trunk		BCC	expression	high
**SK17**	86	female	trunk		BCC	*not detectable*	low
**SK18**	72	male	trunk	pigmented, acanthotic	melanoma; SCC	*not detectable*	low
**SK19**	73	female	trunk		melanoma	*not detectable*	high
**SK20**	35	male	trunk	acanthotic	*no*	*not detectable*	high
**SK21**	75	male	head/neck		melanoma	*not detectable*	high
**SK22**	69	male	trunk	acanthotic	melanoma	*not detectable*	low
**SK23**	66	male	head/neck	irritated (inflamed)	*no*	*not detectable*	low
**SK24**	70	male	head/neck		*no*	expression	high

[a] exome sequencing.

Supplementary Table 4: Primer sequences and conditions (Sanger sequencing)

See Supplementary File 3
