# Supplementary material for: Genetic alterations in seborrheic keratoses
Source: Oncotarget. 2017 Mar 30;8(22):36639–49. doi: 10.18632/oncotarget.16698 (PMC5482683; doi:10.18632/oncotarget.16698)
Supplement: Supplementary file 2 [file oncotarget-08-36639-s002.docx]

| **Supplementary Table 1: Somatic alterations detected through whole-exome sequencing** | | | | | | | | | | |
| --- | --- | --- | --- | --- | --- | --- | --- | --- | --- | --- |
| **Gene Symbol** | **Refseq** | **Chr** | **Position** | **Reference** | **Tumor** | **Context** | **Type** | **Protein**  **Change^a^** | **Mutation**  **Assessor** | **dbSNP** |
| **Single nucleotide variations** | | | | | | | | | | |
| **SAMD11** | **NM_152486** | **chr1** | **878349** | **G** | **A** | **CTGxAGG** | **Missense** | **p.G492E** | **low** |  |
| MORN1 | NM_024848 | chr1 | 2263987 | G | A | ATGxGGG | Intron |  |  |  |
| CAMTA1 | NM_015215 | chr1 | 7527972 | G | A | GCTxGGA | Intron |  |  | rs369914699 |
| MST1L | NM_001271733 | chr1 | 17086217 | C | G | CGAxAGC | Intron |  |  | rs367801802 |
| **HSPG2** | **NM_005529** | **chr1** | **22170735** | **C** | **T** | **TGCxCTT** | **Missense** | **p.G2841E** | **medium** |  |
| RBBP4 | NM_005610 | chr1 | 33149522 | C | T | CCAxCCC | 3'UTR |  |  |  |
| RNF19B | NM_153341 | chr1 | 33412173 | G | A | AGGxGAA | Intron |  |  |  |
| AGO1 | NM_012199 | chr1 | 36385092 | C | T | TTCxTTT | Intron |  |  |  |
| **SCMH1** | **NM_001172219** | **chr1** | **41494363** | **G** | **A** | **GTCxAGA** | **Nonsense** | **p.R584*** |  |  |
| CLDN19 | NM_148960 | chr1 | 43199874 | C | T | TTCxTTA | 3'UTR |  |  |  |
| TIE1 | NM_005424 | chr1 | 43772536 | G | A | CTGxCAT | Nonsense | p.W170* |  |  |
| **SSBP3** | **NM_145716** | **chr1** | **54723760** | **C** | **T** | **CATxATG** | **Missense** | **p.M143I** | **low** |  |
| FPGT-TNNI3K | NM_001112808 | chr1 | 74936803 | C | T | TTCxATC | Intron |  |  |  |
| **COL11A1** | **NM_001854** | **chr1** | **103404641** | **C** | **T** | **TTTxACC** | **Missense** | **p.E1130K^b^** | **low** |  |
| **RNF115** | **NM_014455** | **chr1** | **145663253** | **G** | **A** | **AAAxGGG** | **Missense** | **p.R105K** | **low** |  |
| GON4L | NM_001037533 | chr1 | 155733257 | T | C | TTCxGGC | Silent | p.P1524P |  | rs607834 |
| C1orf226 | NM_001135240 | chr1 | 162355978 | C | T | GGTxTAG | 3'UTR |  |  |  |
| **CFHR5** | **NM_030787** | **chr1** | **196967329** | **G** | **A** | **AAAxAAT** | **Missense** | **p.E348K** | **low** |  |
| SYT2 | NM_177402 | chr1 | 202565418 | C | T | ACAxACA | 3'UTR |  |  |  |
| CNTN2 | NM_005076 | chr1 | 205042389 | C | T | TATxCCC | Intron |  |  |  |
| PFKFB2 | NM_006212 | chr1 | 207245005 | T | C | TTTxATG | Intron |  |  |  |
| ZNF496 | NM_032752 | chr1 | 247463491 | G | A | CGGxGGA | Downstream |  |  |  |
| **OTOF** | **NM_194248** | **chr2** | **26707367** | **C** | **T** | **TCTxATT** | **Missense** | **p.E394K** | **low** |  |
| SLC4A1AP | NM_018158 | chr2 | 27886763 | G | A | TTCxAAG | Silent | p.S48S |  |  |
| LCLAT1 | NM_001002257 | chr2 | 30775483 | G | A | GAAxAGG | Intron |  |  |  |
| LRPPRC | NM_133259 | chr2 | 44114842 | G | A | TTTxAAG | 3'UTR |  |  |  |
| DYSF | NM_001130987 | chr2 | 71744045 | G | A | GAGxGAA | Intron |  |  |  |
| LRP1B | NM_018557 | chr2 | 141474294 | A | G | GCCxTTG | Silent | p.N1950N |  |  |
| SCN2A | NM_001040143 | chr2 | 166248045 | G | A | GCAxTGT | 3'UTR |  |  |  |
| TTN | NM_001256850 | chr2 | 179499208 | G | A | GATxATA | Silent | p.I12459I |  |  |
| **TTN** | **NM_001256850** | **chr2** | **179634844** | **C** | **T** | **CCCxAGC** | **Missense** | **p.G2862R** | **high** |  |
| CASP8 | NM_001080125 | chr2 | 202136494 | G | A | ACAxATG | Intron |  |  |  |
| **PIKFYVE** | **NM_015040** | **chr2** | **209200776** | **G** | **A** | **GAAxAAG** | **Missense** | **p.E1458K** | **neutral** |  |
| **CPS1** | **NM_001875** | **chr2** | **211455539** | **C** | **T** | **GATxGCA** | **Missense** | **p.R286C** | **low** | **rs202130487** |
| ATG16L1 | NM_030803 | chr2 | 234178601 | C | T | TTTxTTA | Intron |  |  |  |
| **SEPT2** | **NM_006155** | **chr2** | **242265453** | **G** | **A** | **GTTxGAT** | **Missense** | **p.G19R** | **medium** |  |
| **FGD5** | **NR_036481** | **chr3** | **14860592** | **C** | **T** | **GTCxGAA** | **Missense** | **p.P5L^a^** | **low** |  |
| PDCD6IP | NM_001162429 | chr3 | 33885745 | G | A | TTTxAAT | Intron |  |  |  |
| PLXNB1 | NM_002673 | chr3 | 48457593 | G | A | AGGxAGA | Intron |  |  |  |
| ARIH2OS | NM_001123040 | chr3 | 48955528 | C | A | CACxGCG | 3'UTR |  |  |  |
| MAGI1 | NM_001033057 | chr3 | 65346537 | C | T | AGTxATT | 3'UTR |  |  |  |
| TRPC1 | NM_001251845 | chr3 | 142499688 | G | A | TGAxGAA | Silent | p.E259E |  |  |
| CLRN1 | NM_052995 | chr3 | 150611194 | G | A | TCGxGCC | UTR |  |  |  |
| LINC01100 (69502bp), C3orf80 (124711bp) | - | chr3 | 159818712 | G | C | GCTxGTC | Intergenic |  |  |  |
| **FGFR3** | **NM_001163213** | **chr4** | **1807890** | **A** | **T** | **AGAxGAC** | **Missense** | **p.K650M** | **low** | **rs121913105** |
| EVC2 | NM_147127 | chr4 | 5570341 | C | T | CCTxGCC | Silent | p.A1129A |  |  |
| EVC2 | NM_147127 | chr4 | 5664824 | G | A | TTAxAAT | Intron |  |  |  |
| EVC | NM_153717 | chr4 | 5806589 | C | T | GTCxCAC | Intron |  |  |  |
| LAP3 | NM_015907 | chr4 | 17598548 | T | G | TGAxCAG | Intron |  |  |  |
| LIMCH1 | NM_014988 | chr4 | 41622715 | C | T | GCTxTGG | Intron |  |  |  |
| PDCL2 | NM_152401 | chr4 | 56458299 | G | A | CAAxAGC | 5'UTR |  |  |  |
| CSN1S2AP | NR_003720 | chr4 | 70938191 | G | A | GTAxAAT | ncRNA_intronic |  |  |  |
| DCK | NM_000788 | chr4 | 71888309 | G | A | TTTxAAG | Intron |  |  |  |
| WDFY3 | NM_014991 | chr4 | 85594105 | G | A | TGAxATT | Silent | p.I3499I |  |  |
| ABCG2 | NM_004827 | chr4 | 89012715 | G | A | AGAxCTA | 3'UTR |  |  |  |
| EIF4E | NM_001130678 | chr4 | 99801975 | G | T | GTGxAAA | 3'UTR |  |  | rs3926191 |
| **QRFPR** | **NM_198179** | **chr4** | **122254184** | **C** | **T** | **GTTxCTT** | **Missense** | **p.E197K** | **neutral** |  |
| POU4F2 | NM_004575 | chr4 | 147563143 | G | A | CAAxGTA | 3'UTR |  |  |  |
| PLRG1 | NM_002669 | chr4 | 155460413 | A | T | AATxATG | Intron |  |  |  |
| F11-AS1 | NR_033900 | chr4 | 187348220 | T | G | TCCxGAT | ncRNA_exonic |  |  |  |
| **EXOC3** | **NM_007277** | **chr5** | **453623** | **G** | **A** | **CGCxTGA** | **Missense** | **p.R168H** | **neutral** | **rs202114916** |
| SLC6A19 | NM_001003841 | chr5 | 1216788 | G | A | GACxACT | Missense | p.D335N | low | rs147646554 |
| MIER3 | NM_152622 | chr5 | 56215718 | G | A | AGTxAAA | 3'UTR |  |  |  |
| PLK2 | NM_006622 | chr5 | 57755955 | G | A | CGGxAAG | 5'UTR |  |  |  |
| **PJA2** | **NM_014819** | **chr5** | **108698676** | **T** | **C** | **TCAxTGT** | **Missense** | **p.N506S^b^** | **low** | **rs139750867** |
| CAMK4 (6518bp), STARD4 (6756bp) | - | chr5 | 110827266 | C | T | TTTxCTA | Intergenic |  |  |  |
| KCNN2 | NM_021614 | chr5 | 113698276 | C | T | AGTxCGC | 5'UTR |  |  | rs116711658 |
| PGGT1B | NM_005023 | chr5 | 114547037 | G | A | ATGxTCG | 3'UTR |  |  |  |
| PCDH1 | NM_032420 | chr5 | 141242659 | C | T | TCCxACT | 3'UTR |  |  |  |
| SH3PXD2B | NM_001017995 | chr5 | 171762021 | A | C | TTTxAGC | 3'UTR |  |  |  |
| **ZKSCAN8** | **NM_006298** | **chr6** | **28120944** | **G** | **A** | **AGGxGTC** | **Missense** | **p.G296S** | **neutral** | **rs62401435** |
| ZSCAN9 | NM_006299 | chr6 | 28199058 | G | A | TGAxACA | Intron |  |  |  |
| KCTD20 | NM_173562 | chr6 | 36442559 | C | T | GTTxTTT | Intron |  |  |  |
| PKHD1 | NM_138694 | chr6 | 51482430 | G | A | TTAxGGA | 3'UTR |  |  |  |
| IL17A | NM_002190 | chr6 | 52054885 | G | A | GAAxAGT | 3'UTR |  |  |  |
| COL9A1 | NM_001851 | chr6 | 70926715 | C | T | ATTxCTG | Missense | p.G884E | high |  |
| MAN1A1 | NM_005907 | chr6 | 119522553 | A | G | AAAxCAA | Intron |  |  | rs9481891 |
| **TAAR2** | **NM_001033080** | **chr6** | **132938597** | **G** | **A** | **TTTxATT** | **Nonsense** | **p.Q250*** | **.** | **rs573651605** |
| LOC100507462 | NM_001242740 | chr6 | 139094641 | C | T | AATxTAA | Intron |  |  |  |
| C6orf99 | NM_001195032 | chr6 | 159291375 | G | A | TCCxAGG | Intron |  |  |  |
| COL28A1 | NM_001037763 | chr7 | 7550797 | G | A | AGAxAAC | Intron |  |  |  |
| DNAH11 | NM_001277115 | chr7 | 21675737 | C | T | ATGxTAT | Intron |  |  |  |
| PDE1C | NM_001191057 | chr7 | 31855584 | C | T | TTTxCCA | Silent | p.G589G |  |  |
| C7orf10 | NM_001193313 | chr7 | 40257157 | C | T | GTTxCTC | Intron |  |  |  |
| POLR2J4 | NM_015983 | chr7 | 43982338 | T | G | CAGxGCG | ncRNA_exonic |  |  |  |
| ZNF92 | NM_152626 | chr7 | 64853867 | G | A | CAAxGAG | Intron |  |  |  |
| HIP1 | NM_005338 | chr7 | 75202978 | G | A | GAAxGAC | Intron |  |  |  |
| EPO | NM_000799 | chr7 | 100320949 | G | A | TAAxGAT | 3'UTR |  |  |  |
| DLD | NM_000108 | chr7 | 107531467 | C | T | ATTxCCA | Upstream |  |  |  |
| CPED1 | NM_024913 | chr7 | 120690811 | C | T | GTGxCCG | Intron |  |  |  |
| **DGKI** | **NM_004717** | **chr7** | **137270005** | **C** | **T** | **TTTxCAC** | **Missense** | **p.E505K** | **low** |  |
| FAM131B | NM_001031690 | chr7 | 143053972 | C | T | ATGxAGG | Missense | p.A224T | neutral |  |
| DPYSL2 | NM_001386 | chr8 | 26514908 | G | A | TAAxCAA | 3'UTR |  |  |  |
| IMPAD1 | NM_017813 | chr8 | 57871694 | C | T | ATAxTTA | 3'UTR |  |  |  |
| CSMD3 | NM_198123 | chr8 | 113516241 | G | A | AAAxAGA | Intron |  |  |  |
| CSMD3 | NM_198123 | chr8 | 113516247 | T | A | AAGxAGG | Intron |  |  | rs371162285 |
| DERL1 | NM_024295 | chr8 | 124054557 | C | T | TCCxCTT | 5'UTR |  |  |  |
| **TBC1D31** | **NM_145647** | **chr8** | **124121624** | **C** | **T** | **ATCxAAC** | **Missense** | **p.P426L** | **medium** |  |
| HSF1 | NM_005526 | chr8 | 145535111 | G | A | CAGxGCT | Intron |  |  |  |
| TMEM261 | NM_033428 | chr9 | 7796710 | G | A | GAAxATG | 3'UTR |  |  |  |
| IZUMO3 | NM_001271706 | chr9 | 24543436 | G | T | ATTxTTT | Intron |  |  | rs33972842 |
| **SYK** | **NM_003177** | **chr9** | **93624601** | **G** | **A** | **AGGxAAA** | **Missense** | **p.G231E** | **low** |  |
| C9orf91 | NM_153045 | chr9 | 117406517 | C | T | ATCxCCA | 3'UTR |  |  |  |
| RALGPS1 | NM_014636 | chr9 | 129946378 | G | A | CAGxAGG | Intron |  |  | rs538775479 |
| **PRKCQ** | **NM_006257** | **chr10** | **6470173** | **G** | **T** | **CAGxATA** | **Missense** | **p.S706Y** | **neutral** |  |
| SFMBT2 | NM_001018039 | chr10 | 7203538 | C | T | TGCxCGG | 3'UTR |  |  |  |
| **CUBN** | **NM_001081** | **chr10** | **16946114** | **C** | **T** | **TCAxCTG** | **Splice_Site** | **p.G2638D** | **medium** | **rs150330148** |
| **ZNF37A** | **NM_003421** | **chr10** | **38407643** | **C** | **T** | **AAAxCCT** | **Missense** | **p.P522S** | **medium** |  |
| MARCH8 | NM_145021 | chr10 | 45959789 | C | T | AGAxCTG | Intron |  |  |  |
| LRRC20 (6856bp), EIF4EBP2 (14599bp) | - | chr10 | 72149262 | G | A | CCAxAAT | Intergenic |  |  |  |
| USP54 | NM_152586 | chr10 | 75290091 | C | T | GGTxCTA | Silent | p.R546R**^a^** |  |  |
| IDE | NM_004969 | chr10 | 94216053 | A | C | GCTxTGA | Intron |  |  |  |
| **WDR96** | **NM_025145** | **chr10** | **105942155** | **G** | **A** | **ACGxAAA** | **Missense** | **p.S684F** | **low** |  |
| SORCS1 | NM_001013031 | chr10 | 108447907 | C | T | CATxCCT | Intron |  |  |  |
| C10orf90 | NM_001004298 | chr10 | 128209933 | C | A | GGTxTTT | 5'UTR |  |  |  |
| **KIAA1549L** | **NM_012194** | **chr11** | **33596335** | **A** | **T** | **GGGxCCG** | **Missense** | **p.T1143S** | **low** |  |
| MS4A2 | NM_000139 | chr11 | 59863149 | C | T | ACAxTCT | 3'UTR |  |  |  |
| CD6 | NM_006725 | chr11 | 60785957 | G | A | AGGxGAA | Intron |  |  |  |
| CPT1A | NM_001876 | chr11 | 68523681 | C | T | ACTxTGA | 3'UTR |  |  |  |
| ARRB1 | NM_004041 | chr11 | 74998337 | C | T | CTCxTCT | Intron |  |  |  |
| MOGAT2 | NM_025098 | chr11 | 75442425 | C | T | AGCxTTC | Downstream |  |  |  |
| DGAT2 | NM_032564 | chr11 | 75509389 | C | T | TTTxGCC | Silent | p.F309F |  | rs143158001 |
| **RNF214** | **NM_207343** | **chr11** | **117152823** | **C** | **T** | **TCCxTTG** | **Missense** | **p.L517F** | **low** |  |
| BCL9L | NM_182557 | chr11 | 118767283 | C | T | GCCxTGG | 3'UTR |  |  |  |
| **C11orf63** | **NM_199124** | **chr11** | **122775901** | **C** | **T** | **TCTxTCC** | **Missense** | **p.L296F** | **neutral** |  |
| CLEC1B | NM_016509 | chr12 | 10151825 | G | A | TGAxATG | 5'UTR |  |  |  |
| GOLT1B | NM_016072 | chr12 | 21670615 | G | A | AAAxGGT | 3'UTR |  |  |  |
| **CDK2** | **NM_001798** | **chr12** | **56362709** | **C** | **T** | **GTCxCTG** | **Missense** | **p.P155S** | **low** |  |
| MON2 | NM_015026 | chr12 | 62981932 | G | A | TGAxAAT | Silent | p.E1662E |  |  |
| E2F7 | NM_203394 | chr12 | 77424139 | G | A | GAAxTTA | Intron |  |  |  |
| MKRN9P | NR_033410 | chr12 | 88178201 | C | T | TGCxGCG | ncRNA_exonic |  |  |  |
| RAD9B | NM_152442 | chr12 | 110940010 | C | A | AGCxGTT | 5'UTR |  |  |  |
| RNFT2 | NM_001109903 | chr12 | 117288990 | C | A | TTCxAAC | Intronic |  |  |  |
| SUDS3 | NM_022491 | chr12 | 118854592 | G | A | TTTxAAG | 3'UTR |  |  |  |
| CAMKK2 | NM_006549 | chr12 | 121706544 | G | A | AGGxAGG | Intron |  |  |  |
| **LRRC43** | **NM_152759** | **chr12** | **122677439** | **G** | **A** | **TCGxAGC** | **Missense** | **p.E413K** | **low** |  |
| FLT1 | NM_002019 | chr13 | 28876695 | G | A | AAGxGTC | 3'UTR |  |  |  |
| **USPL1** | **NM_005800** | **chr13** | **31231683** | **C** | **T** | **CTTxAGA** | **Missense** | **p.S490L** | **medium** |  |
| CCDC169 | NM_001198910 | chr13 | 36871851 | C | T | TTCxTTC | Silent | p.K2K |  |  |
| LRCH1 | NM_015116 | chr13 | 47273710 | C | G | TGTxGTG | Intron |  |  |  |
| **OLFM4** | **NM_006418** | **chr13** | **53624856** | **G** | **A** | **AACxATG** | **Missense** | **p.D495N^b^** | **low** |  |
| **DIAPH3** | **NM_001042517** | **chr13** | **60384922** | **C** | **T** | **CACxAGT** | **Splice_Site** | **p.E1055K** | **medium** |  |
| OXGR1 | NM_080818 | chr13 | 97639159 | G | A | AACxATG | Silent | p.I285I**^a^** |  |  |
| NALCN | NM_052867 | chr13 | 101717955 | G | A | CTCxATG | Intron |  |  |  |
| NPAS3 | NM_001164749 | chr14 | 34273334 | C | T | TTTxATC | 3'UTR |  |  |  |
| NIN | NM_020921 | chr14 | 51232977 | C | T | GGTxTGA | Intron |  |  |  |
| AKT1 | NM_001014431 | chr14 | 105237053 | G | A | GGAxTGG | Intron |  |  |  |
| AHNAK2 | NM_138420 | chr14 | 105413144 | G | A | CTGxGAG | Missense | p.P2882S | medium |  |
| TJP1 | NM_003257 | chr15 | 29991829 | A | G | CATxGAT | 3'UTR |  |  |  |
| TJP1 | NM_003257 | chr15 | 29996537 | G | A | AGAxAGG | Intron |  |  | rs375658941 |
| CHAC1 | NM_024111 | chr15 | 41248424 | G | A | AGGxCAT | 3'UTR |  |  |  |
| JMJD7-PLA2G4B | NM_001198588 | chr15 | 42129801 | T | C | TTTxTTT | Intron |  |  |  |
| GALK2 | NM_002044 | chr15 | 49621716 | G | C | TGCxCAA | 3'UTR |  |  |  |
| **NEDD4** | **NM_001284338** | **chr15** | **56125281** | **C** | **T** | **TTTxTGA** | **Missense** | **p.E1237K** | **medium** |  |
| IGDCC3 | NM_004884 | chr15 | 65624070 | G | A | ATGxAGA | Intron |  |  |  |
| LINC00052 (285446bp), NTRK3 (11625bp) | - | chr15 | 88408363 | G | A | CTAxGAT | Intergenic |  |  |  |
| PIGQ | NM_148920 | chr16 | 628746 | G | A | TGGxGTC | Intron |  |  |  |
| ARL6IP1 | NM_015161 | chr16 | 18812646 | G | A | AGAxAAG | Intron |  |  |  |
| SMG1 | NM_015092 | chr16 | 18849664 | G | A | GAAxAGA | Intron |  |  |  |
| GDE1 | NM_016641 | chr16 | 19522082 | A | T | AAAxTAT | Intron |  |  |  |
| RRN3P1 | NR_003370 | chr16 | 21817392 | T | C | AAAxGAA | ncRNA_intronic |  |  | rs370774778 |
| MYO1C | NM_001080779 | chr17 | 1368130 | G | A | GACxGAG | 3'UTR |  |  |  |
| SLC13A5 | NM_177550 | chr17 | 6610216 | C | T | TGTxTCC | Intron |  |  |  |
| NTN1 | NM_004822 | chr17 | 9146324 | C | T | TACxAGA | 3'UTR |  |  |  |
| CCT6B | NM_006584 | chr17 | 33285759 | G | A | AAGxAAG | Intron |  |  |  |
| **HEATR6** | **NM_022070** | **chr17** | **58136846** | **G** | **A** | **GACxATC** | **Missense** | **p.R554C** | **medium** |  |
| LRRC37A3 (9991bp), AMZ2P1 (37774bp) | - | chr17 | 62924894 | G | T | CTAxGAT | Intergenic |  |  |  |
| ABCA9 | NM_080283 | chr17 | 66987117 | G | A | TGTxATA | Intron |  |  |  |
| **MIB1** | **NM_020774** | **chr18** | **19429183** | **C** | **T** | **CTTxTAT** | **Missense** | **p.S807F** | **neutral** |  |
| **SH2D3A** | **NM_005490** | **chr19** | **6754323** | **G** | **A** | **CCTxGCC** | **Missense** | **p.P404L** | **low** |  |
| ZNF559 | NM_001202412 | chr19 | 9449312 | G | T | GTAxATG | Intron |  |  |  |
| DOCK6 | NM_020812 | chr19 | 11348278 | G | A | GACxAGG | Intron |  |  | rs573427255 |
| **ARRDC2** | **NM_015683** | **chr19** | **18119357** | **G** | **A** | **GTGxAGG** | **Missense** | **p.E80K** | **low** |  |
| UPF1 | NM_002911 | chr19 | 18966764 | C | T | CATxGCC | Silent | p.I536I |  |  |
| NDUFA13 | NM_015965 | chr19 | 19638774 | A | G | TATxTGG | Intron |  |  |  |
| **IGFLR1** | **NM_024660** | **chr19** | **36231982** | **G** | **A** | **CTGxGTT** | **Missense** | **p.P34S** | **low** |  |
| **CD79A** | **NM_001783** | **chr19** | **42383670** | **G** | **A** | **GAGxGGA** | **Missense** | **p.G149R** | **low** |  |
| **RTN2** | **NM_005619** | **chr19** | **45997610** | **C** | **T** | **AGCxCGG** | **Missense** | **p.G210S** | **neutral** |  |
| GYS1 | NM_002103 | chr19 | 49477582 | C | T | CTCxGGG | Silent | p.P479P |  |  |
| PRKCG | NM_002739 | chr19 | 54387368 | G | A | GCTxGAC | Intron |  |  |  |
| LILRA5 | NM_021250 | chr19 | 54818963 | G | A | ACTxAGA | Intron |  |  |  |
| PCSK2 | NM_002594 | chr20 | 17463945 | G | A | TGAxAGA | 3'UTR |  |  |  |
| RALGAPB | NM_020336 | chr20 | 37153692 | G | A | ATAxTCC | Intron |  |  |  |
| ATP9A | NM_006045 | chr20 | 50238562 | G | A | ATAxGAC | Intron |  |  |  |
| **EEF1A2** | **NM_001958** | **chr20** | **62127325** | **C** | **G** | **TGCxGCG** | **Missense** | **p.G70R** | **high** |  |
| MYT1 | NM_004535 | chr20 | 62871269 | C | T | ACGxGGG | Intron |  |  | rs371337079 |
| **GRIK1** | **NM_000830** | **chr21** | **30971238** | **G** | **A** | **CCAxTCA** | **Missense** | **p.T373I** | **medium** |  |
| HUNK | NM_014586 | chr21 | 33372910 | C | T | CAAxTTT | 3'UTR |  |  |  |
| **HIRA** | **NM_003325** | **chr22** | **19398286** | **G** | **A** | **ACTxAAA** | **Missense** | **p.S18L^b^** | **high** |  |
| YPEL1 | NM_013313 | chr22 | 22053020 | G | T | CCAxCTT | 3'UTR |  |  |  |
| BMS1P20 (53442bp), ZNF280B (108006bp) | - | chr22 | 22730766 | C | T | CTTxTCT | Intergenic |  |  |  |
| NCF4 | NM_000631 | chr22 | 37260265 | C | T | ATTxTCT | Intron |  |  |  |
| CYB5R3 | NM_000398 | chr22 | 43015525 | G | A | ATGxGGC | 3'UTR |  |  |  |
| MPPED1 | NM_001044370 | chr22 | 43894235 | G | A | TAAxGCT | Intron |  |  |  |
| **BCOR** | **NM_001123385** | **chrX** | **39922959** | **G** | **A** | **TTAxTCC** | **Missense** | **p.T1250I** | **neutral** | **rs144795138** |
| SHROOM4 | NM_020717 | chrX | 50341515 | G | A | GCTxATG | Silent | p.I1321I |  |  |
| BCYRN1 | NR_001568 | chrX | 70586067 | C | T | CCCxCTC | ncRNA_intronic |  |  |  |
| CHIC1 | NM_001039840 | chrX | 72783021 | C | T | ACAxCCC | 5'UTR |  |  |  |
| **RPS6KA6** | **NM_014496** | **chrX** | **83319312** | **C** | **T** | **TTTxATG** | **Missense** | **p.M737I** | **low** |  |
| GPRASP1 | NM_014710 | chrX | 101908967 | C | T | GACxCAG | Silent | p.T42T**^b^** |  |  |
| COL4A5 | NM_000495 | chrX | 107940425 | C | T | GCCxCCT | 3'UTR |  |  |  |
| XIAP | NM_001167 | chrX | 123046951 | G | A | AGGxACT | 3'UTR |  |  |  |
| **Dinucleotide variations:** | | | | | | | | | | |
| HES2 | NM_019089 | chr1 | 6477921 | G | A | ACAxGCG | 3'UTR |  |  |  |
| HES2 | NM_019089 | chr1 | 6477922 | G | A | CAGxCGT | 3'UTR |  |  |  |
| **MFSD2A** | **NM_001136493** | **chr1** | **40433321** | **C** | **T** | **ATTxCCA** | **Missense** | **p.P358F** | **medium** |  |
| **MFSD2A** | **NM_001136493** | **chr1** | **40433322** | **C** | **T** | **TTCxCAT** | **Missense** |  |  |  |
| **SLC39A1** | **NM_001271960** | **chr1** | **153935010** | **C** | **T** | **GAGxCTT** | **Missense** | **p.G61B** | **neutral** |  |
| **SLC39A1** | **NM_001271960** | **chr1** | **153935011** | **C** | **T** | **AGCxTTC** | **Missense** |  |  |  |
| **PRCC** | **NM_005973** | **chr1** | **156756880** | **C** | **T** | **GCCxCTT** | **Missense** | **p.P333F** | **neutral** |  |
| **PRCC** | **NM_005973** | **chr1** | **156756881** | **C** | **T** | **CCCxTTG** | **Missense** |  |  |  |
| KLHL30 ^c^ | NM_198582 | chr2 | 239060003 | G | T | GCCxGGG | 3'UTR |  |  |  |
| KLHL30 | NM_198582 | chr2 | 239060004 | G | A | CCGxGGG | 3'UTR |  |  |  |
| CSPG5 | NM_001206943 | chr3 | 47621891 | C | T | CCTxCGG | Upstream |  |  |  |
| CSPG5 | NM_001206943 | chr3 | 47621892 | C | T | CTCxGGG | Upstream |  |  | rs116718919 |
| **ST6GAL1** | **NM_003032** | **chr3** | **186760722** | **C** | **T** | **GACxCTC** | **Silent** | **p.T77T** |  |  |
| **ST6GAL1** | **NM_003032** | **chr3** | **186760723** | **C** | **T** | **ACCxTCG** | **Missense** | **p.L78F** | **medium** | **rs140438545** |
| **SEL1L3** | **NM_015187** | **chr4** | **25759216** | **C** | **T** | **TTCxCAG** | **Missense** | **p.G1067R** | **medium** |  |
| **SEL1L3** | **NM_015187** | **chr4** | **25759217** | **C** | **T** | **TCCxAGA** | **Silent** | **p.L1066L** |  |  |
| PSD2 | NM_032289 | chr5 | 139202232 | C | T | TCTxCTG | Intron |  |  |  |
| PSD2 | NM_032289 | chr5 | 139202233 | C | T | CTCxTGA | Intron |  |  |  |
| CAMK2B | NM_001220 | chr7 | 44302475 | G | A | GTGxGAT | Intron |  |  |  |
| CAMK2B | NM_001220 | chr7 | 44302476 | G | A | TGGxATA | Intron |  |  |  |
| GPR26 | NM_153442 | chr10 | 125447956 | G | A | CCTxGGG | 3'UTR |  |  |  |
| GPR26 | NM_153442 | chr10 | 125447957 | G | A | CTGxGGG | 3'UTR |  |  |  |
| SLC35C1 | NM_018389 | chr11 | 45833327 | G | A | AGAxGGT | 3'UTR |  |  |  |
| SLC35C1 | NM_018389 | chr11 | 45833328 | G | A | GAGxGTT | 3'UTR |  |  |  |
| SPATA19 | NM_174927 | chr11 | 133714248 | G | A | GAGxGTC | Intron |  |  |  |
| SPATA19 | NM_174927 | chr11 | 133714249 | G | A | AGGxTCC | Intron |  |  |  |
| **HOXC13** | **NM_017410** | **chr12** | **54333067** | **G** | **A** | **TCGxGGG** | **Missense** | **p.G126E** | **medium** |  |
| **HOXC13** | **NM_017410** | **chr12** | **54333068** | **G** | **A** | **CGGxGGC** | **Silent** |  |  |  |
| ATP11A (22875bp), MCF2L-AS1 (57441bp) | - | chr13 | 113564357 | G | A | AAAxGAT | Intergenic |  |  |  |
| ATP11A (22876), MCF2L-AS1 (57440) | - | chr13 | 113564358 | G | A | AAGxATA | Intergenic |  |  |  |
| **DLST** | **NM_001933** | **chr14** | **75365173** | **C** | **T** | **TTGxCTT** | **Missense** | **p.A291V** | **medium** |  |
| **DLST** | **NM_001933** | **chr14** | **75365174** | **C** | **T** | **TGCxTTG** | **Silent** |  |  |  |
| CNGB1 | NM_001297 | chr16 | 57994670 | G | A | GGAxGAG | Intron |  |  |  |
| CNGB1 | NM_001297 | chr16 | 57994671 | G | A | GAGxAGG | Intron |  |  |  |
| ZDHHC7 | NM_017740 | chr16 | 85008492 | G | A | TTCxGAT | 3'UTR |  |  | rs112925578 |
| ZDHHC7 | NM_017740 | chr16 | 85008493 | G | A | TCGxATG | 3'UTR |  |  |  |
| RPH3AL | NM_001190411 | chr17 | 63581 | G | A | GGAxGAG | 3'UTR |  |  |  |
| RPH3AL | NM_001190411 | chr17 | 63582 | G | A | GAGxAGC | 3'UTR |  |  |  |
| **C17orf74** | **NM_175734** | **chr17** | **7330419** | **C** | **T** | **AACxCTT** | **Missense** | **p.P370L** | **neutral** |  |
| **C17orf74** | **NM_175734** | **chr17** | **7330420** | **C** | **T** | **ACCxTTG** | **Silent** |  |  |  |
| ZNF20 | NM_001098507 | chr19 | 12242836 | G | A | ACAxGCT | 3'UTR |  |  |  |
| ZNF20 | NM_001098507 | chr19 | 12242837 | G | A | CAGxCTC | 3'UTR |  |  |  |
| TAF4 | NM_003185 | chr20 | 60585244 | G | A | CAAxGTA | Intron |  |  |  |
| TAF4 | NM_003185 | chr20 | 60585245 | G | A | AAGxTAA | Intron |  |  |  |
| **MTG2** | **NM_015666** | **chr20** | **60773872** | **C** | **T** | **TCTxCAC** | **Silent** | **p.L217L** |  |  |
| **MTG2** | **NM_015666** | **chr20** | **60773873** | **C** | **T** | **CTCxACC** | **Missense** | **p.H218Y** | **neutral** | **rs371962931** |
| LOC400891 | NR_027006 | chr22 | 21414818 | C | T | TGAxCTT | ncRNA_exonic |  |  |  |
| LOC400891 | NR_027006 | chr22 | 21414819 | C | T | GACxTTG | ncRNA_exonic |  |  |  |
| PRPS2 | NM_001039091 | chrX | 12828395 | C | T | AGAxCGC | Intron |  |  |  |
| PRPS2 | NM_001039091 | chrX | 12828396 | C | T | GACxGCA | Intron |  |  |  |
| **ARHGAP4** | **NM_001666** | **chrX** | **153178681** | **G** | **A** | **GAAxGGC** | **Missense** | **p.L468F** | **low** |  |
| **ARHGAP4** | **NM_001666** | **chrX** | **153178682** | **G** | **A** | **AAGxGCC** | **Silent** | **p.A467A** |  |  |
| **Trinucleotide variation** | | | | | | | | | | |
| **AQP11** ^c^ | **NM_173039** | **chr11** | **77314638** | **G** | **T** | **TTTxGCA** | **Missense** | **p.L219F** | **medium** |  |
| **AQP11** ^c^ | **NM_173039** | **chr11** | **77314639** | **G** | **A** | **TTGxCAC** | **Missense** | **p.A220K** |  |  |
| **AQP11** | **NM_173039** | **chr11** | **77314640** | **C** | **A** | **TGGxACT** | **Missense** | **p.A220K** |  |  |
| **Insertion** | | | | | | | | | | |
| **WDR44** | **NM_019045** | **chrX** | **117526907** | **-** | **AG** | **TTA[AG]ATG** | **Frameshift** |  |  |  |
| ^a^Amino acid annotated for the transcript with amino acid changes  ^b^ Variant reported in COSMIC  ^c^Variant not called by Mutect, discovered during manual reviewing.  Assessment of mutation type was performed by ANNOVAR.  Mutations validated by Sanger sequencing are **in bold**. | | | | | | | | | | |
