# Supplementary material for: Genetic alterations in seborrheic keratoses
Source: Oncotarget. 2017 Mar 30;8(22):36639–49. doi: 10.18632/oncotarget.16698 (PMC5482683; doi:10.18632/oncotarget.16698)
Supplement: Supplementary file 3 [file oncotarget-08-36639-s003.docx]

| **Supplementary Table 2: Validated somatic variants detected by Sanger sequencing** | | | | | | | |
| --- | --- | --- | --- | --- | --- | --- | --- |
| **Chr** | **Start ^a^** | **End ^a^** | **Ref** | **Tumor** | **Protein Change** | **Gene** | **Gene Description** |
| **Single nucleotide variations** | | | | | | | |
| chr1 | 878349 | 878349 | G | A | p.G492E | SAMD11 | sterile alpha motif domain containing 11 |
| chr1 | 22170735 | 22170735 | C | T | p.G2841E | HSPG2 | heparan sulfate proteoglycan 2 |
| chr1 | 41494363 | 41494363 | G | A | p.R584X | SCMH1 | sex comb on midleg homolog 1 (Drosophila) |
| chr1 | 54723760 | 54723760 | C | T | p.M143I | SSBP3 | single stranded DNA binding protein 3 |
| chr1 | 103404641 | 103404641 | C | T | p.E1130K | COL11A1 | collagen, type XI, alpha 1 |
| chr1 | 145663253 | 145663253 | G | A | p.R105K | RNF115 | ring finger protein 115 |
| chr1 | 196967329 | 196967329 | G | A | p.E348K | CFHR5 | complement factor H-related 5 |
| chr2 | 26707367 | 26707367 | C | T | p.E394K | OTOF | otoferlin |
| chr2 | 179634844 | 179634844 | C | T | p.G2862R | TTN | titin |
| chr2 | 209200776 | 209200776 | G | A | p.E1458K | PIKFYVE | phosphoinositide kinase, FYVE finger containing |
| chr2 | 211455539 | 211455539 | C | T | p.R286C | CPS1 | carbamoyl-phosphate synthase 1, mitochondrial |
| chr2 | 242265453 | 242265453 | G | A | p.G19R | SEPT2 | septin 2 |
| chr3 | 14860592 | 14860592 | C | T | p.P5L | FGD5 | FYVE, RhoGEF and PH domain containing 5 |
| chr4 | 1807890 | 1807890 | A | T | p.K650M | FGFR3 | fibroblast growth factor receptor 3 |
| chr4 | 122254184 | 122254184 | C | T | p.E197K | QRFPR | pyroglutamylated RFamide peptide receptor |
| chr5 | 453623 | 453623 | G | A | p.R168H | EXOC3 | exocyst complex component 3 |
| chr5 | 108698676 | 108698676 | T | C | p.N506S | PJA2 | praja ring finger 2, E3 ubiquitin protein ligase |
| chr6 | 28120944 | 28120944 | G | A | p.G296S | ZKSCAN8 | zinc finger with KRAB and SCAN domains 8 |
| chr6 | 132938597 | 132938597 | G | A | p.Q250X | TAAR2 | trace amine associated receptor 2 |
| chr7 | 137270005 | 137270005 | C | T | p.E505K | DGKI | diacylglycerol kinase, iota |
| chr8 | 124121624 | 124121624 | C | T | p.P426L | TBC1D31 | TBC1 domain family, member 31 |
| chr9 | 93624601 | 93624601 | G | A | p.G231E | SYK | spleen tyrosine kinase |
| chr10 | 6470173 | 6470173 | G | T | p.S706Y | PRKCQ | protein kinase C, theta |
| chr10 | 16946114 | 16946114 | C | T | p.G2638D | CUBN | cubilin (intrinsic factor-cobalamin receptor) |
| chr10 | 38407643 | 38407643 | C | T | p.P522S | ZNF37A | zinc finger protein 37A |
| chr10 | 105942155 | 105942155 | G | A | p.S684F | WDR96 | WD Repeat-Containing Protein 96 |
| chr11 | 33596335 | 33596335 | A | T | p.T1143S | KIAA1549L | KIAA1549-Like |
| chr11 | 117152823 | 117152823 | C | T | p.L517F | RNF214 | ring finger protein 214 |
| chr11 | 122775901 | 122775901 | C | T | p.L296F | C11orf63 | chromosome 11 open reading frame 63 |
| chr12 | 56362709 | 56362709 | C | T | p.P155S | CDK2 | cyclin-dependent kinase 2 |
| chr12 | 122677439 | 122677439 | G | A | p.E413K | LRRC43 | leucine rich repeat containing 43 |
| chr13 | 31231683 | 31231683 | C | T | p.S490L | USPL1 | ubiquitin specific peptidase like 1 |
| chr13 | 53624856 | 53624856 | G | A | p.D495N | OLFM4 | olfactomedin 4 |
| chr13 | 60384922 | 60384922 | C | T | p.E1055K | DIAPH3 | diaphanous-related formin 3 |
| chr15 | 56125281 | 56125281 | C | T | p.E1237K | NEDD4 | neural precursor cell expressed, developmentally down-regulated 4,  E3 ubiquitin protein ligase |
| chr17 | 58136846 | 58136846 | G | A | p.R554C | HEATR6 | HEAT repeat containing 6 |
| chr18 | 19429183 | 19429183 | C | T | p.S807F | MIB1 | mindbomb E3 ubiquitin protein ligase 1 |
| chr19 | 6754323 | 6754323 | G | A | p.P404L | SH2D3A | SH2 domain containing 3A |
| chr19 | 18119357 | 18119357 | G | A | p.E80K | ARRDC2 | arrestin domain containing 2 |
| chr19 | 36231982 | 36231982 | G | A | p.P34S | IGFLR1 | IGF-like family receptor 1 |
| chr19 | 42383670 | 42383670 | G | A | p.G149R | CD79A | CD79a molecule, immunoglobulin-associated alpha |
| chr19 | 45997610 | 45997610 | C | T | p.G210S | RTN2 | reticulon 2 |
| chr20 | 62127325 | 62127325 | C | G | p.G70R | EEF1A2 | eukaryotic translation elongation factor 1 alpha 2 |
| chr21 | 30971238 | 30971238 | G | A | p.T373I | GRIK1 | glutamate receptor, ionotropic, kainate 1 |
| chr22 | 19398286 | 19398286 | G | A | p.S18L | HIRA | histone cell cycle regulator |
| chrX | 39922959 | 39922959 | G | A | p.T1250I | BCOR | BCL6 corepressor |
| chrX | 83319312 | 83319312 | C | T | p.M737I | RPS6KA6 | ribosomal protein S6 kinase, 90kDa, polypeptide 6 |
| **Dinucleotide variants** | | | | | | | |
| chr1 | 40433321 | 40433322 | CC | TT | p.P358F | MFSD2A | major facilitator superfamily domain containing 2A |
| chr1 | 153935010 | 153935011 | CC | TT | p.G61B | SLC39A1 | solute carrier family 6 (neutral amino acid transporter), member 19 |
| chr1 | 156756880 | 156756881 | CC | TC | p.P333F | PRCC | papillary renal cell carcinoma (translocation-associated) |
| chr3 | 186760722 | 186760723 | CC | TT | p.T77T, p.L78F | ST6GAL1 | ST6 beta-galactosamide alpha-2,6-sialyltranferase 1 |
| chr4 | 25759216 | 25759217 | CC | TT | p.G1067R, p.L1066L | SEL1L3 | sel-1 suppressor of lin-12-like 3 (C. elegans) |
| chr12 | 54333067 | 54333068 | GG | AA | p.G126E | HOXC13 | homeobox C13 |
| chr14 | 75365173 | 75365174 | CC | TT | p.A291V | DLST | dihydrolipoamide S-succinyltransferase (E2 component of 2-oxo-glutarate complex) |
| chr17 | 7330419 | 7330420 | CC | TT | p.P370L | C17orf74 | chromosome 17 open reading frame 74 |
| chr20 | 60773872 | 60773873 | CC | TT | p.L217L, p.H218Y | MTG2 | mitochondrial ribosome-associated GTPase 2 |
| chrX | 153178681 | 153178682 | GG | AA | p.L468F, p.A467A | ARHGAP4 | Rho GTPase activating protein 4 |
| **Trinucleotide variant** | | | | | | | |
| chr11 | 77314638 | 77314640 | GGC | TAA | p.L219F, p.A220K | AQP11 | aquaporin 11 |
| **Insertion** | | | | | | | |
| chrX | 117526907 | 117526908 | - | AG | p.N16*fs*X174 | WDR44 | WD Repeat Domain 44 |
| ^a^ Genome reference: hg19; 1-based coordinates | | | | | | | |
