# Supplementary material for: Genetic alterations in seborrheic keratoses
Source: Oncotarget. 2017 Mar 30;8(22):36639–49. doi: 10.18632/oncotarget.16698 (PMC5482683; doi:10.18632/oncotarget.16698)
Supplement: Supplementary file 4 [file oncotarget-08-36639-s004.docx]

| **Supplementary Table 4: Primer sequences and conditions (Sanger sequencing)** | | | | |
| --- | --- | --- | --- | --- |
| **Gene** | **Primer** | **Primer Sequence 5' --> 3'** | **Ta** |  |
| *AGHAPA* | AGHAPA-F | CCAGGGGTCTTCAAGGCATC | 53 | 2.5 mM MgCl2, 5% Glycerol |
|  | AGHAPA-R | CTAACACTTGGGGCTCTTCTTGG |  |  |
| *AQP11* | AQP11-F | GCCAGGTGATTCTGCAAAGGAAG | 53 | 2.5 mM MgCl2, 5% Glycerol |
|  | AQP11-R | GTGCCTGCTGTGGCTTTG |  |  |
| *ARRDC2* | ARRDC2-F | CGTCGAGCCCGTGTTTAGCG | 56 | 2.5 mM MgCl2, 5% DMSO |
|  | ARRDC2-R | CCAGGGCTGACGGAGGTAC |  |  |
| *BCOR* | BCOR-F | GGATGACCCACCCTCCAGGAG | 57 | 2.5 mM MgCl2, 5% Glycerol |
|  | BCOR-R | CGCCACTTGCTGCACCTTAGAG |  |  |
| *C11orf63* | C11orf63-F | GTGGGTAGGGTACAACATAAGG | 50 | 2.5 mM MgCl2 |
|  | C11orf63-R | GGACTCTACCATTCTAACCCC |  |  |
| *C17orf74* | C17orf74-F | CTGCCTCGGTGTCCCGGAACG | 60 | 2.0 mM MgCl2, 5% DMSO |
|  | C17orf74-R | GCTTGGAGCTGGGGTCCGTTG |  |  |
| *CD79A* | CD79A-F | GCTCTGAGCCATACTACCTCCTTG | 54 | 2.5 mM MgCl2 |
|  | CD79A-R | CTAGAGGGCTACCTGGGTATTGG |  |  |
| *CDK2* | CDK2-F | CATCGGGTCCTCCACCGAGAC | 57 | 2.5 mM MgCl2 |
|  | CDK2-R | GTGGGCCCAGTTAGCAACACAG |  |  |
| *CFHR5* | CFHR5-F | CCAACAGCATTGTCTATTTTGTGC | 50 | 2.5 mM MgCl2 |
|  | CFHR5-R | CTGTGCAGTCTACTTCAGGATTC |  |  |
| *COL11A1* | COL11A1-F | CTCCCTTGTCACCCTTGC | 51 | 2.5 mM MgCl2 |
|  | COL11A1-R | CTAGAAGCAGTGGGAACCCA |  |  |
| *CPS1* | CPS1-F | GGGACCCATAAGACCTGCAGG | 53 | 2.5 mM MgCl2 |
|  | CPS1-R | CCTCACCTGTTGGCCATGGAC |  |  |
| *CUBN* | CUBN-F | GTTCTACACGTTCGTTGGTGAC | 51 | 2.5 mM MgCl2, 10% Glycerol |
|  | CUBN-R | CCCTTGGAGGGACTTTCTG |  |  |
| *DGKI* | DGKI-F | GCTCAGGCAGATGCTCCAGGG | 59 | 1.5 mM MgCl2 |
|  | DGKI-R | ACGGCTTGGCTCTTCCTTGCG |  |  |
| *DIAPH3* | DIAPH3-F | CTACTCGTTAGTAAATGGAAGGG | 48 | 2.0 mM MgCl2 |
|  | DIAPH3-R | CATTTTGCGGGATGAATCACTGG |  |  |
| *DLST* | DLST-F | GTAACATCCAGGAGATGAGGGCTC | 54 | 2.5 mM MgCl2, 5% Glycerol |
|  | DLST-R | GCTGCTTCAGGAAGGTGAACC |  |  |
| *EEF1A2* | EEF1A2-F | CCGGGGGCATCGATGATGGTG | 59 | 2.5 mM MgCl2, 5% Glycerol |
|  | EEF1A2-R | CACCCGCTCCAGATGGGGAAG |  |  |
| *EXOC3* | EXOC3-F | GCACTCCTGCAAGCCCACCG | 61 | 2.5 mM MgCl2 |
|  | EXOC3-R | CAGCAAGGTGGGGTCACGGC |  |  |
| *FGD5* | FGD5-F | GGATGGACGGAACCATCTGTGG | 56 | 2.5 mM MgCl2 |
|  | FGD5-R | GAACCCTGGGGACCACGATG |  |  |
| *GRIK1* | GRIK1-F | GGAGGTTACCTTTTCAGTTCCTTC | 51 | 2.5 mM MgCl2 |
|  | GRIK1-R | GTCGCTGAACCAATGCAATGG |  |  |
| *HEATR6* | HEATR6-F | GCCATCAGTTTTAAGCCACTAC | 49 | 2.5 mM MgCl2, 5% Glycerol |
|  | HEATR6-R | CTATGCTGTGGTTTAATCAAGCC |  |  |
| *HIRA* | HIRA-F | GCAAGGTGGCAGGGCTGTTAAG | 57 | 2.5 mM MgCl2, 5% Glycerol |
|  | HIRA-R | CAGGCATCCTATGGGCTCAGC |  |  |
| *HOXC13* | HOXC13-F | CTCAGGGCGCCGTCTATACG | 55 | 2.5 mM MgCl2, 5% DMSO |
|  | HOXC13-R | CCTAGAGGACAGGTCGTCACC |  |  |
| *HSPG2* | HSPG2-F | GGATGCATGCATCCTTCTCCAG | 54 | 2.5 mM MgCl2, 5% Glycerol |
|  | HSPG2-R | CACTGACCTCTCCCCTGTGC |  |  |
| *IGFLR1* | IGFLR1-F | GCTCCCTATCACCTGCAAGC | 54 | 2.5 mM MgCl2 |
|  | IGFLR1-R | GGAAAGTGTAGCAGAGGCGC |  |  |
| *KIAA1549L* | KIAA1549L-F | GGTGAAGATGCAGCGTGTCCC | 56 | 2.5 mM MgCl2 |
|  | KIAA1549L-R | CCCTTACGGTCAGCTTGCAGAAG |  |  |
| *LRRC43* | LRRC43-F | CAGACACCCCCGGCATCAGC | 60 | 2.5 mM MgCl2 |
|  | LRRC43-R | TCCTTACCCTGGGGACGGGC |  |  |
| *MFSD2A* | MFSD2A-F | CAGGGCTGGGAGCCACTTTG | 56 | 2.5 mM MgCl2, 5% Glycerol |
|  | MFSD2A-R | CTCTCCATGAGGGCCACCAAG |  |  |
| *MIB1* | MIB1-F | GTATCTAAAGATGCAGAGACGC | 48 | 2.5 mM MgCl2 |
|  | MIB1-R | GAGGCATTTCTTGACACGTG |  |  |
| *MTG2* | MTG2-F | GCCGACCTGTCTTGCGTGGG | 60 | 2.5 mM MgCl2, 5% Glycerol |
|  | MTG2-R | GCTGGGGGAGTCACAAGGGC |  |  |
| *NEDD4* | NEDD4-F | GGAAGCTTGCGGATTCGTCAGC | 56 | 2.5 mM MgCl2, 5% DMSO |
|  | NEDD4-R | CCTCTTTCCCAGTGCTGTTCCC |  |  |
| *OLFM4* | OLFM4-F | GGTATGTGGGGTTCTGTATGC | 50 | 2.5 mM MgCl2 |
|  | OLFM4-R | CTATGCACCTAAACATTGCTGC |  |  |
| *OTOF* | OTOF-F | GGGTGGCAGGTGCTCTCAGC | 59 | 2.5 mM MgCl2, 5% Glycerol |
|  | OTOF-R | TGTGACGTTGCCGTGGTGGG |  |  |
| *PIKFYVE* | PIKFYVE-F | GTTGCCATTGATGAAAGACTTGC | 49 | 2.5 mM MgCl2 |
|  | PIKFYVE-R | CACACCTGTTATTCCAAGCTTG |  |  |
| *PJA2* | PJA2-F | CACACTGGAGTCATCCTCC | 50 | 2.5 mM MgCl2, 5% DMSO |
|  | PJA2-R | CTAGGGAACAGACATCCTTGG |  |  |
| *PRCC* | PRCC-F | CACCTGGAGTTGAGCCATACC | 52 | 2.5 mM MgCl2, 5% Glycerol |
|  | PRCC-R | GACCACCATGTTCCAACATTCC |  |  |
| *PRKCQ* | PRKCQ-F | GTCACATGGGGGCGAACGGG | 60 | 2.5 mM MgCl2 |
|  | PRKCQ-R | AGCCCCGGCTGTCATTTGCC |  |  |
| *QRFPR* | QRFPR-F | GCATCACCATAAGAGGCAGGAG | 52 | 2.5 mM MgCl2 |
|  | QRFPR-R | CTCATTTGAAATACGTGCTGGGG |  |  |
| *RNF115* | RNF115-F | CTTTGGGGCCATTTGGATCACAC | 54 | 2.5 mM MgCl2 |
|  | RNF115-R | AGCTGGAGATCTGTCAGGACG |  |  |
| *RNF214* | RNF214-F | CCATGGTTATGCCCAGTGCAG | 55 | 2.5 mM MgCl2 |
|  | RNF214-R | GGTCAGCAGCTTCTCCAGGATC |  |  |
| *RPS6KA6* | RPS6KA6-F | GCAGACAACGATGCCTTTG | 50 | 2.5 mM MgCl2 |
|  | RPS6KA6-R | GGAGCAATGGTTGCAACATAC |  |  |
| *RTN2* | RTN2-F | CGACTCCGACTTGCTCAGCCC | 59 | 2.5 mM MgCl2, 10% Glycerol |
|  | RTN2-R | CTCTCGCTCCAGTGGCCCC |  |  |
| *SAMD11* | SAMD11-F | GCGAGTCCAAGGAGATGACGG | 56 | 1.5 mM MgCl2 |
|  | SAMD11-R | GTCTCCTGACCGTCGTGTGC |  |  |
| *SCMH1* | SCMH1-F | CCTACTGAGAGGTCAGGCAGC | 55 | 2.5 mM MgCl2 |
|  | SCMH1-R | GCCACTGAGATGTGCCAAGG |  |  |
| *SEL1L3* | SEL1L3-F | CCTGAGACAGACTGGAAATACTGC | 53 | 2.5 mM MgCl2, 5% Glycerol |
|  | SEL1L3-R | GCTGGAGCCACAGTAACGAG |  |  |
| *SEPT2* | SEPT2-F | GAAGTATGACCTTCTGGAAGTG | 48 | 2.0 mM MgCl2, 10% DMSO |
|  | SEPT2-R | CAGAGATTGGAAAGGGGAG |  |  |
| *SH2D3A* | SH2D3A-F | CAGTCTTCCCAGTCACCCGCAC | 59 | 1.5 mM MgCl2 |
|  | SH2D3A-R | CCAGACCCTTGCCCTCCACC |  |  |
| *SLC39A1* | SLC39A1-F | CCTTTCCCTCCTCCTTTGGTCAC | 56 | 2.5 mM MgCl2, 5% Glycerol |
|  | SLC39A1-R | GTAGCTTCAGAGCCTCCAGTGC |  |  |
| *SSBP3* | SSBP3-F | GACTCGCAGGCAGTGGCTAG | 57 | 2.5 mM MgCl2 |
|  | SSBP3-R | GCTTCTGCGCTTCTCCATGTGC |  |  |
| *ST6GAL1* | ST6GAL1-F | GCAAACCAAGGAATTCCAGGTG | 52 | 2.5 mM MgCl2 |
|  | ST6GAL1-R | CTGCACTGAACTTGATGCCTGG |  |  |
| *SYK* | SYK-F | GCCTGCTGCACGAAGGGAAGG | 60 | 2.5 mM MgCl2, 5% Glycerol |
|  | SYK-R | CCCACGTGCCCTGTACTCTACCC |  |  |
| *TAAR2* | TAAR2-F | GTACTTCAGTGCTCTGCGAAACC | 54 | 2.5 mM MgCl2, 5% Glycerol |
|  | TAAR2-R | CAGTTCCTGCCCAGTGATGTTC |  |  |
| *TBC1D31* | TBC1D31-F | GTTGGGAAGTCACTGTGGAC | 50 | 2.5 mM MgCl2 |
|  | TBC1D31-R | CTATGAGGGTACTAAACGCAGTATG |  |  |
| *TTN* | TTN-F | TGCCTGATACTTACTCTCCACAAACAG | 54 | 2.5 mM MgCl2, 5% Glycerol |
|  | TTN-R | GCCAAGTGACAAACACAGACTGG |  |  |
| *USPL1* | USPL1-F | GGCCAGAAGAAAGTTAACCTAGG | 53 | 2.5 mM MgCl2 |
|  | USPL1-R | CATCACCCACAGAACACGATG |  |  |
| *WDR44* | WDR44-F | CTGCCAGTCCTATTGTGGCTAG | 53 | 2.5 mM MgCl2, 5% Glycerol |
|  | WDR44-R | GAGTCTGAGGACACAGGCTC |  |  |
| *WDR96* | WDR96-F | CTCTCAGACGTAGCTTATTCCC | 50 | 2.5 mM MgCl2 |
|  | WDR96-R | GCGATTTGGAGGACACCTAG |  |  |
| *ZKSCAN8* | ZKSCAN8-F | GGTGGTGAGACCAGGAGTGAG | 55 | 2.5 mM MgCl2 |
|  | ZKSCAN8-R | CAGTGTGGATTCTCCAGTGGCG |  |  |
| *ZNF37A* | ZNF37A-F | CGTCAGAAGTCAGCCCTAATTG | 50 | 2.5 mM MgCl2, 5% Glycerol |
|  | ZNF37A-R | CTCCCCAAGTGTATTCTCTGATG |  |  |
| *BRAF* | BRAF-exon15-F | CCTAAACTCTTCATAATGCTT | 51 | 2.5 mM MgCl2 |
|  | BRAF-exon15-R | ATGGTAAGAATTGAGGCTAT |  |  |
| *CDKN2A* | CDKN2A-exon1-F | CGGCTGCGGAGAGGGGGAGAG | 56 | 2.5 mM MgCl2, 5% Glycerol |
|  | CDKN2A-exon1-R | GGATGGCGGGCGACTCTGGAG |  |  |
|  | CDKN2A-exon2-F | TTTGGAAGCTCTCAGGGTACA | 52 | 2.0 mM MgCl2 |
|  | CDKN2A-exon2-R | GGGCTCTACACAAGCTTCCTT |  |  |
|  | CDKN2A-exon3-F | ATGCCGGTAGGGACGGCAAG | 58 | 2.5 mM MgCl2, 7.5% Glycerol |
|  | CDKN2A-exon3-R | AAAGCGGGGTGGGTTGTGGC |  |  |
| *DPH3* promoter | DPH3p-F | 5'CGAAGGGGTAACGCCCCAG3' | 56 | 2.5 mM MgCl2, 5% DMSO |
|  | DPH3p-R | 5'GGTCCCAGACGTGACGTAGC3' |  |  |
| *FGFR3* | FGFR3-exon10-F | GGCATCCATGGGAGCCCCG | 61 | 2.5 mM MgCl2 |
|  | FGFR3-exon10-R | GACGAGGAGAGGGGAGCCCG |  |  |
|  | FGFR3-exon15-F | CACCTTCAAGGACCTGGTGT | 60 | 2.0 mM MgCl2, 10 % Glycerol |
|  | FGFR3-exon15-R | TGTACGTGTCCTGCAGAGCT |  |  |
|  | FGFR3-exon7-F | GAGAACAAGTTTGGCAGCATC | 60 | 2.0 mM MgCl2, 10 % Glycerol |
|  | FGFR3-exon7-R | CAAGGTGTACAGTGACGCACAG |  |  |
| *FOXN1* | FOXN1-exon1-F | CTGGTCCTCACTCTCATGGCAGACG | 57 | 2.5 mM MgCl2 |
|  | FOXN1-exon1-R | CCGGGTACTTACACTCTGTGGGG |  |  |
|  | FOXN1-exon2-F | CTCTCTGTCTACCCAGAAGCATGCC | 57 | 2.5 mM MgCl2 |
|  | FOXN1-exon2-R | GCCACTAGTACTCACCAGGACTTGGG |  |  |
|  | FOXN1-exon3-F | GTAAGGTTCAAGACACAGGAAAGAG | 51 | 2.5 mM MgCl2 |
|  | FOXN1-exon3-R | CCAGACCCACCTGGTGGAAG |  |  |
|  | FOXN1-exon4-F | GGTACCATGCAATCACTCTGCC | 53 | 2.5 mM MgCl2 |
|  | FOXN1-exon4-R | CTGGAGAGTGGGAAATGTACCTG |  |  |
|  | FOXN1-exon5-F | GTCAGAGGTTCGGACTCTCAGG | 54 | 2.5 mM MgCl2 |
|  | FOXN1-exon5-R | GGAGGAATCTTGGGCTCACC |  |  |
|  | FOXN1-exon6-F | GCTTCCTGAGCCTGGCCTGAATG | 59 | 2.5 mM MgCl2 |
|  | FOXN1-exon6-R | CCTGGGCCCTTCCTTGCGTG |  |  |
|  | FOXN1-exon7-F | GGAGGGAGGTCTCATGGTGTTC | 55 | 2.5 mM MgCl2 |
|  | FOXN1-exon7-R | CCAGCTCACCCTGGAAGTCG |  |  |
|  | FOXN1-exon8-F | CCTCCCAGTGACACCTGTTCTCTCC | 59 | 2.5 mM MgCl2 |
|  | FOXN1-exon8-R | CGCTGGAGCTGACGAAGCTGG |  |  |
| *HRAS* | HRAS-exon1-F | GAGGAGCGATGACGGAATATAAGCTG | 55 | 2.5 mM MgCl2 |
|  | HRAS-exon1-R | CAATGACCACCTGCTTCCGGTAG |  |  |
|  | HRAS-exon2-F | GGAAGCAGGTGGTCATTGATG | 52 | 2.5 mM MgCl2, 10% DMSO |
|  | HRAS-exon2-R | CTTCACCCGTTTGATCTGCTC |  |  |
| *PIK3CA* | PIK3CA-exon10-F | CTGGATTTGTTCTACAAATATTATG | 50 | 1.5 mM MgCl2 |
|  | PIK3CA-exon10-R | GTAAATTCTGCTTTATTTATTCC |  |  |
|  | PIK3CA-exon21-F | GCTCCAAACTGACCAAACTGTTC | 52 | 2.0 mM MgCl2, 5% DMSO |
|  | PIK3CA-exon21-R | CAGTGCAGTGTGGAATCCAG |  |  |
| *TERT* promoter | TERTp-27-F | 5'CCCACGTGCGCAGCAGGAC3' | 60 | 2 mM MgCl2, 5% Glycerol |
|  | TERTp-286-R | 5'CTCCCAGTGGATTCGCGGGC3' |  |  |
